# Ibrutinib and rituximab versus immunochemotherapy in patients with previously untreated mantle cell lymphoma (ENRICH): a randomised, open-label, phase 2/3 superiority trial

**DOI:** 10.1016/S0140-6736(25)01432-1

**Published:** 2025-10-25

**Authors:** David J Lewis, Mats Jerkeman, Lexy Sorrell, David Wright, Ingrid Glimelius, Christian B Poulsen, Annika Pasanen, Andrew Rawstron, Karin F Wader, Nick Morley, Catherine Burton, Andrew J Davies, Ingemar Lagerlöf, Surita Dalal, Ruth De Tute, Chris McNamara, Nicola Crosbie, Helle Erbs Toldbod, Jeanette Sanders, Victoria Allgar, Sree Aroori, Mark Warner, Claire Scully, Brian Wainman, Jacob Haaber Christensen, Jon Riise, Kristina Sonnevi, Mark J Bishton, Toby A Eyre, Simon Rule, ENRICH investigators, David Lewis, Simon Rule, Mark Bishton, Toby Eyre, Chris McNamara, Nick Morley, Michelle Furtado, Adam Forbes, Cathy Burton, Pam McKay, Nimish Shah, Andrew Davies, Meghna Ruparelia, Jo Addada, Shankara Paneesha, Andrew Hodson, Deborah Turner, Fiona Miall, Marian Macheta, Renata Walewska, Clare Rowntree, Sunil Iyengar, Kim Linton, Rebecca Auer, Moya Young, Arvind Arumainathan, Santosh Narat, James Milnthorpe, Jonathan Cullis, Iain Singer, Annika Whittle, Dominic Culligan, Rory McCulloch, Richard Lush, Angus Broom, Mohsen Norouzi, Sudarshan Gurung, Maadh Aldouri, Marketa Wilson, Piers Patten, Yasmin Hasan, Paul Kerr, Beth Harrison, Peter Forsyth, Moez Dungarwalla, Unmesh Mohite, Oonagh Sheehy, Wendy Osborne, Gavin Campbell, Russell Patmore, Vikram Singh, Fiona Cutler, Paul Micallef-Eynaud, Rachel Boulton-Jones, Ruth Pettengell, Ingrid Glimelius, Mats Jerkeman, Kristina Sonnevi, Ingemar Lagerlöf, Lena Brandefors, Nevzeta Kuric, Catharina Lewerin, Karin Papworth, Jon Riise, Karin Wader, Jacob Haaber Christensen, Christian Bjørn Poulsen, Hans Herluf Nørregaard Bentzen, Pär Lars Josefsson, Annika Pasanen, Marjukka Pollari

**Affiliations:** a Department of Haematology, University Hospitals Plymouth NHS Trust, Plymouth, UK; b Department of Oncology, Skane University Hospital, Lund, Sweden; c Peninsula Medical School, University of Plymouth, Plymouth, UK; d Department of Clinical and Biomedical Sciences, University of Exeter, Exeter, Devon, UK; e Department of Immunology, Genetics and Pathology, Uppsala University, Uppsala, Sweden; f Zealand University Hospital Roskilde, Roskilde, Denmark; g Department of Oncology, Helsinki University Hospital Comprehensive Cancer Center, Helsinki, Finland; h Haematological Malignancy Diagnostic Service, Leeds Cancer Centre, Leeds Teaching Hospitals NHS Trust, Leeds, UK; i St Olav's Hospital HF, Trondheim, Norway; j Department of Haematology, Sheffield Teaching Hospitals NHS Foundation Trust, Sheffield, UK; k Cancer Sciences Division, University of Southampton, Southampton, UK; l Linköping University Hospital, Linköping, Sweden; m Department of Haematology, University College London, London, UK; n Aarhus University Hospital, Aarhus, Denmark; o Odense University Hospital, Odense, Denmark; p Oslo University Hospital, Oslo, Norway; q Department of Haematology, Karolinska University Hospital, Stockholm; r Academic Unit of Translational Medical Sciences, University of Nottingham, Nottingham, UK; s Haematology and Cancer Centre, Churchill Hospital, Oxford University Hospitals NHS Trust, Oxford, UK; t AstraZeneca, Cambridge, UK

## Abstract

**Background:**

Ibrutinib, a Bruton tyrosine kinase inhibitor, prolongs progression-free survival when added to immunochemotherapy as first line treatment. The ENRICH trial compared the chemotherapy-free combination of ibrutinib and the anti-CD20 antibody rituximab (ibrutinib–rituximab) with standard immunochemotherapy (R-CHOP [rituximab–cyclophosphamide, doxorubicin, vincristine, and prednisolone] or bendamustine–rituximab) in patients 60 years and older with untreated mantle-cell lymphoma.

**Methods:**

This randomised, open-label, phase 2/3 superiority trial was performed at 66 sites in the UK, Sweden, Norway, Finland, and Denmark. Patients 60 years and older with untreated mantle-cell lymphoma (Ann–Arbor stage II–IV disease, an Eastern Cooperative Oncology Group performance-status score of 0–2) were randomly assigned to receive either rituximab plus immunochemotherapy or ibrutinib–rituximab in a 1:1 ratio, stratified by investigator choice of immunochemotherapy. Patients randomly allocated to the ibrutinib–rituximab (intervention) group received 560 mg oral ibrutinib daily in combination with six to eight cycles of 375 mg/m^2^ intravenous rituximab on day 1 of each cycle in the matched schedule of the pre-randomisation choice of immunochemotherapy (every 21 days for R-CHOP or every 28 days for rituximab–bendamustine). R-CHOP comprised 750 mg/m^2^ of cyclophosphamide, 50 mg/m^2^ of doxorubicin, and 1·4 mg/m^2^ vincristine on day 1 of each 21-day cycle, with 100 mg prednisolone on days 1–5 of each cycle. Rituximab–bendamustine comprised 90 mg/m^2^ of bendamustine on days 1 and 2 of each cycle, in combination with 375 mg/m^2^ rituximab on day 1 of each cycle. All responding patients in both groups at the end of induction received maintenance rituximab administered every 8 weeks for 2 years, and patients allocated to the intervention group continued ibrutinib until disease progression or unacceptable toxicity. The primary outcome was investigator-assessed progression-free survival, stratified by immunochemotherapy choice and analysed in the intention-to-treat population. The trial was registered with EudraCT (2015–000832–13) and is closed for recruitment.

**Findings:**

Between Feb 15, 2016, and June 30, 2021, 397 patients were randomly allocated to immunochemotherapy (control) or ibrutinib–rituximab (intervention). Of the 397, 107 (27%) were pre-allocated to the immunochemotherapy choice of R-CHOP and 290 (73%) were pre-allocated to rituximab–bendamustine. In total, 198 were allocated to the control group (53 to R-CHOP and 145 to bendamustine–rituximab) and 199 were allocated to intervention. The median age was 74 years (IQR 70–77) for the intervention group and 74 years (70–78) in the control group. 296 patients (75%) were male and 101 patients (25%) were female; ethnicity data were not collected. At a median follow-up of 47·9 months, the median progression-free survival of ibrutinib–rituximab was superior to immunochemotherapy, with an adjusted hazard ratio (HR) of 0·69 (95% CI 0·52–0·90); p=0·0034. For those with pre-randomisation choice R-CHOP, the HR was 0·37 (0·22–0·62), and with bendamustine–rituximab, the HR was 0·91 (0·66–1·25). Across induction and maintenance, 67% of patients assigned to ibrutinib–rituximab and 70% of patients receiving immunotherapy reported grade 3 or above adverse events.

**Interpretation:**

To our knowledge, this is the first randomised trial in untreated mantle-cell lymphoma to demonstrate significant improvement in progression-free survival for ibrutinib–rituximab compared to immunochemotherapy. This study suggests that ibrutinib–rituximab should be considered a new standard of care option for first-line treatment of older patients with mantle-cell lymphoma.

**Funding:**

Cancer Research UK (C7627/A17938) and Johnson and Johnson Pharmaceuticals.

**Research in context**

**Evidence before this study**

We searched PubMed from database inception to Jan 10, 2025, without language restrictions, for randomised phase 3 studies comparing the role of Bruton's tyrosine inhibitors with immunochemotherapy in the treatment of older patients with mantle cell lymphoma who were not eligible for transplantation. We used the search terms (“mantle cell lymphoma” OR “MCL”) AND (“Phase 3” OR “Phase III” OR “Randomized”) AND (“ibrutinib” OR “BTKi”); this produced 65 results, of which two were randomised studies in first-line treatments of mantle cell lymphoma. The SHINE trial, which compared bendamustine–rituximab–ibrutinib with bendamustine–rituximab, demonstrated an improvement in progression-free survival but not overall survival with the addition of ibrutinib to immunochemotherapy. In patients aged 18–65 years, the TRIANGLE trial has demonstrated an improvement in failure-free survival with the addition of ibrutinib to standard high-dose chemotherapy. No published phase 3 trial has compared ibrutinib–rituximab to immunochemotherapy.

**Added value of this study**

The ENRICH trial demonstrated a significant improvement in progression-free survival for patients 60 years and older with mantle cell lymphoma treated with ibrutinib–rituximab compared with standard immunochemotherapy approaches. This was primarily due to an improvement in progression-free survival compared to R-CHOP (rituximab–cyclophosphamide, doxorubicin, vincristine, and prednisolone with rituximab–bendamustine) chemotherapy.

**Implications of all the available evidence**

Ibrutinib–rituximab should be regarded as a standard of care option for the first line treatment of older patients with mantle cell lymphoma. This study supports the use of Bruton tyrosine kinase inhibitors in the first-line treatment of mantle-cell lymphoma. and will provide a benchmark for future trials.

## Introduction

Mantle-cell lymphoma is an uncommon subtype of B-cell lymphoma characterised by the chromosomal translocation t(11;14)(q13;q32). The disease is considered incurable with biological features such as morphological subtype, proliferation rate and *TP53* mutations influencing the risk of early relapse.^1^ Therapeutic options for older patients with untreated mantle-cell lymphoma include R-CHOP (rituximab–cyclophosphamide, doxorubicin, vincristine, and prednisolone) followed by maintenance rituximab, and VR-CAP (bortezomib, rituximab, cyclophosphamide, doxorubicin, and prednisone).^2^, ^3^, ^4^, ^5^ Randomised trials without maintenance have shown rituximab–bendamustine improves progression-free survival compared to R-CHOP, and the addition of rituximab maintenance improves progression-free survival and overall survival after R-CHOP. Observational data also suggest improved progression-free survival for rituximab maintenance following rituximab–bendamustine.^4^, ^6^, ^7^ VR-CAP has demonstrated improved overall survival compared with R-CHOP, but the trial was performed without maintenance rituximab and VR-CAP was associated with excess haematological, neurological, and gastrointestinal toxicity.^2^ In population datasets the most commonly used regimens in patients who are ineligible for transplantation are rituximab–bendamustine and R-CHOP.^8^

Covalent oral Bruton tyrosine kinase (BTK) inhibitors (ibrutinib, zanubrutinib, and acalabrutinib) have superior response rates, duration of response, and progression-free survival as second line treatment compared to when it is used as a later treatment and compared to immunochemotherapy.^9^, ^10^, ^11^, ^12^, ^13^, ^14^ Adding ibrutinib to the induction phase of R-CHOP alternating with R-DHAP (rituximab, dexamethasone, cytarabine, and cisplatin) improves failure-free survival and overall survival compared to standard induction and autologous stem-cell transplantation (ASCT), but is considered too intensive for patients older than 65 years.^15^, ^16^ In older patients the addition of ibrutinib to rituximab–bendamustine improves progression-free survival at the expense of increased non-lymphoma related deaths, leading to no benefit in overall survival.^17^ Particular toxicities leading to early cessation of ibrutinib include cardiac toxicity (particularly atrial fibrillation), haemorrhage, and infection.^12^, ^17^, ^18^ Single-arm trials have demonstrated the efficacy of ibrutinib–rituximab in untreated mantle-cell lymphoma,^19^ however ibrutinib–rituximab has never previously been compared with immunochemotherapy in a first-line randomised controlled trial.

Here we report the primary results of the international randomised phase 2/3 ENRICH trial, which aimed to demonstrate the superiority of the combination of ibrutinib–rituximab versus immunochemotherapy (investigator choice of R-CHOP or rituximab–bendamustine), each with maintenance rituximab, in patients 60 years or older with untreated mantle-cell lymphoma.

## Methods

### Study design and participants

ENRICH is an investigator-lead, multicentre, randomised, open-label superiority trial. Patients were recruited from 66 university hospitals in the UK, Sweden, Norway, Finland, and Denmark. Ethical approval was obtained from the ethics committees of all participating countries (appendix p 2).

We enrolled previously untreated patients 60 years and older with histologically confirmed mantle-cell lymphoma, Ann–Arbor stage II–IV disease, an Eastern Cooperative Oncology Group (ECOG) performance-status score of 0–2, at least one measurable lesion by CT, and who required treatment. Exclusion criteria included patients with planned ASCT, or if patients presented with CNS involvement. Complete eligibility criteria are provided in the trial protocol (appendix). Gender (options of male or female) data were recorded by investigators using medical records. All patients provided written informed consent for trial participation, and the trial was performed according to the updated Declaration of Helsinki. Tumour histology was reported at local sites, including assessment of proliferation using Ki67 expression. The trial was registered with EudraCT (2015–000832–13) and is closed for recruitment.

### Randomisation and masking

Patients were randomly assigned to receive either rituximab plus immunochemotherapy or ibrutinib–rituximab in a 1:1 ratio, stratified by investigator choice of immunochemotherapy (R-CHOP or rituximab–bendamustine). The randomisation system was prepared in conjunction with a statistician who had no other role in the trial. Randomisation was performed using a web-based data entry system once all eligibility assessment and consent processes was completed. As an open-label design, no masking was done.

### Procedures

During the treatment induction phase, patients randomly allocated to the ibrutinib–rituximab (intervention) group received 560 mg oral ibrutinib daily in combination with six to eight cycles of 375 mg/m^2^ intravenous rituximab on day 1 of each cycle in the matched schedule of the pre-randomisation choice of immunochemotherapy (every 21 days for R-CHOP or every 28 days for rituximab–bendamustine). Induction for the control group consisted of six to eight cycles of R-CHOP or rituximab–bendamustine immunochemotherapy. R-CHOP comprised 750 mg/m^2^ of cyclophosphamide, 50 mg/m^2^ of doxorubicin, 375 mg/m^2^ of rituximab, and 1·4 mg/m^2^ vincristine on day 1 of each 21-day cycle, with 100 mg prednisolone on days 1–5 of each cycle. Rituximab–bendamustine comprised 90 mg/m^2^ of bendamustine on days 1 and 2 of each cycle (which was defined as 28 days), in combination with 375 mg/m^2^ rituximab on day 1 of each cycle. At the mid-induction assessment, patients in the intervention group with stable disease were permitted to continue treatment to allow for slower responses to ibrutinib. For patients with a response to treatment the maintenance phase consisted of 12 cycles of 8-weekly 1400 mg subcutaneous rituximab on day 1 of each cycle for both groups, with the addition of daily ibrutinib in the intervention group. During follow-up, ibrutinib was continued until disease progression, unacceptable toxicity, death, or patient decision. Crossover was not permitted during the trial.

Response assessments were recorded according to the investigator at each site. Clinical examinations were performed at each visit. CT scans of the neck, chest, abdomen, and pelvis were performed at baseline; mid-induction treatment; end of induction treatment; maintenance visits 3, 6, and 9; and at the end of maintenance. Subsequent imaging was performed at investigator discretion. Bone marrow evaluation was performed at baseline. For patients with bone marrow involvement, evaluations were also done at mid-induction, end of induction, and end of maintenance. Next generation sequencing for pathogenic *TP53* mutations was performed on available baseline biopsies in a central laboratory.

### Outcomes

The primary endpoint was investigator-assessed progression-free survival, defined as the time from random allocation to disease progression or death from any cause, as determined by investigator assessment.

Secondary endpoints were overall survival, defined as the time from random allocation to death; objective response (assessed in accordance with the International Workshop Standardised Response Criteria for Non-Hodgkin lymphoma and radiologically assessed by CT criteria);^20^ safety and toxicity; quality of life; and time to next treatment, defined as the time from random allocation to the next mantle-cell lymphoma treatment or death.

Safety was assessed up to 30 days after the last dose of trial treatment. Prespecified adverse events included cardiac, bleeding, and infectious events of grade 3 or above. Adverse events were classified according to the National Cancer Institute Common Terminology Criteria for Adverse Events version 4.03.

### Statistical analysis

The planned sample size was 400 participants to detect superiority of ibrutinib–rituximab versus immunochemotherapy, assuming a recruitment rate of 100 patients per year, a follow-up period of 3 years after the final patient was randomly allocated, a predicted loss to follow-up at a rate of 0·05 per year, a hazard ratio (HR) of 0·67 with a 30-month median progression-free survival in the immunochemotherapy group, and a statistical power of 90%. For further details see the statistical analysis plan (appendix). Participants who were lost to follow-up or withdrew from participation in the trial were censored at the time of last recorded contact.

An interim phase 2 analysis was performed on April 18, 2019**,** to assess overall response rate in the ibrutinib–rituximab group, with a required overall response rate of 75% or above to continue recruitment and the phase 3 trial. Results were reviewed by a Data Monitoring Committee and Trial Steering Committee, who recommended continuation of the trial into phase 3.

The primary analysis was a test of superiority of ibrutinib–rituximab versus immunochemotherapy at the one-sided 2·5% level for progression-free survival. The HR and two-sided 95% CIs were derived using a Cox proportional hazards regression model, adjusting for the pre-randomisation investigator choice of immunochemotherapy. Due to the possibility of a difference between the two immunochemotherapy regimens, the primary analysis included a prespecified assessment of the treatment effect stratified by the choice of immunochemotherapy. Consistency of the treatment effect was measured by a test of interaction between allocation and pre-randomisation choice of immunochemotherapy. The consistency of the treatment effect was evaluated across prespecified subgroups of interest, including age group, ECOG, blastoid disease status, and Mantle Cell Lymphoma International Prognostic Index (MIPI) score. Additional subgroup analyses were conducted for gender, disease status, Ki67 over 30%, and *TP53* mutation.

Secondary survival endpoints, including overall survival and time to next treatment, were analysed using Cox regression models. Objective response was summarised according to the best response per patient, and quality-of-life (measured by EORTC QLQ-C30) was summarised descriptively. All efficacy endpoints were analysed in the intention-to-treat population, while safety was assessed in the population that completed at least one cycle of treatment.

Additional pre-planned analyses were conducted for the outcomes of progression-free survival and overall survival on the per-protocol population, and a separate sensitivity analysis censoring COVID-19 deaths at the participant's last trial visit and failure-free survival (modified progression-free survival, including next mantle-cell lymphoma treatment and stable disease at mid-induction).

Adverse events were evaluated according to treatment periods (induction and maintenance treatment). Safety was compared between the ibrutinib–rituximab intervention group and the R-CHOP and rituximab–bendamustine control groups. Adverse events were classified as haematological if the MedDRA preferred term included thrombocytopenia, neutropenia, anaemia, febrile neutropenia, platelet count decreased, white blood cell count decreased, neutropenic infection, neutropenic sepsis, neutrophil count decreased, haemoglobin decreased, lymphocyte count decreased, or lymphopenia.

A Data Monitoring Committee supervised the progress of the trial and ensured patient safety, trial data, and scientific integrity. All analyses were conducted using R version 4.4.0.

### Role of the funding source

The funders of the trial had no role in trial design, data collection, data analysis, data interpretation, or writing the Article.

## Results

**Figure 1:**
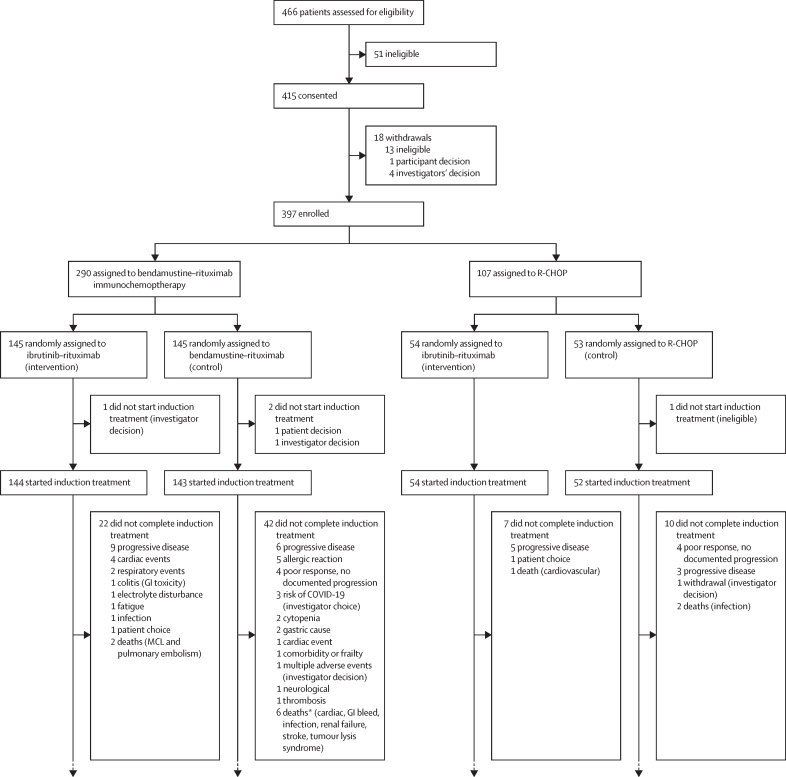

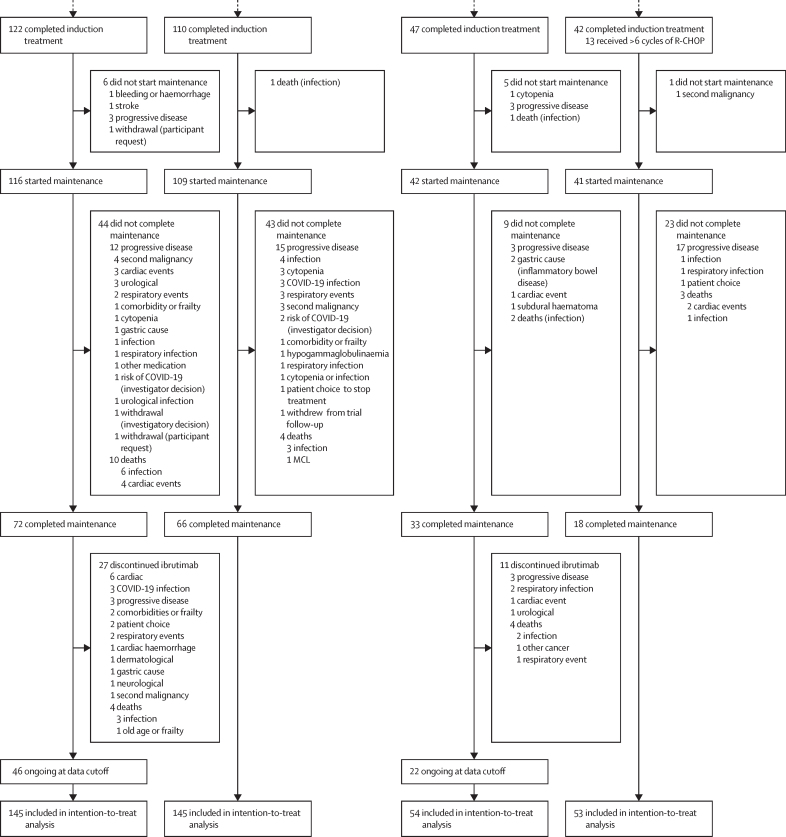
ENRICH trial profile The trial profile details the ENRICH treatment pathway for participants. Not all progressions of disease or deaths are listed, as events might occur once a participant has discontinued ENRICH treatment (appendix pp 32, 36). GI=gastrointestinal. MCL=mantle-cell lymphoma. R-CHOP=rituximab–cyclophosphamide, doxorubicin, vincristine, and prednisolone. *One death for each named cause.

Between Feb 15, 2016, and June 30, 2021, 466 patients from the UK, Sweden, Norway, Finland, and Denmark were screened for eligibility; of these 415 were eligible and after 18 patients did not proceed to random allocation. 397 patients were randomly allocated to immunochemotherapy (control) or ibrutinib–rituximab (intervention). Of the 397, 107 (27%) were pre-allocated to the immunochemotherapy choice of R-CHOP and 290 (73%) were pre-allocated to rituximab–bendamustine. In total, 198 were allocated to the control group and 199 were allocated to intervention (figure 1, appendix p 3). The median age was 74 years (IQR 70–77) for the intervention group and 74 years (70–78) in the control group. 296 patients (75%) were male and 101 patients (25%) were female; ethnicity data was not collected as ethnicity data are illegal to collect in Sweden. 222 (56%) of 393 had a high MIPI score; four patients had missing data (table 1). Of the patients with available data, 25 (7%) of 370 had blastoid disease (appendix pp 27–28). Participants who were lost to follow-up or withdrew from the study were censored at the time of last recorded contact. At the data cutoff point for the primary analysis on June 30, 2024, the median follow-up was 47·9 months (IQR 27·1–63·3).

**Table 1:** Baseline participant characteristics by pre-randomisation investigator choice of immunochemotherapy and treatment allocation of the intention-to-treat population

		**Bendamustine–rituximab**		**R-CHOP**		**Overall**	
		Bendamustine–rituximab (n=145)	Ibrutinib–rituximab (n=145)	R-CHOP (n=53)	Ibrutinib– rituximab (n=54)	Immunochemotherapy (n=198)	Ibrutinib– rituximab (n=199)
Age, years							
	Median (IQR)	74 (71–78)	74 (70–77)	72 (69–76)	74 (70–77)	74 (70–78)	74 (70–77)
	60–69	27 (19%)	36 (25%)	18 (34%)	15 (28%)	45 (23%)	51 (26%)
	70–79	96 (66%)	94 (65%)	33 (62%)	35 (65%)	129 (65%)	129 (65%)
	80–89	22 (15%)	15 (10%)	2 (4%)	4 (7%)	24 (12%)	19 (10%)
Gender							
	Male	107 (74%)	109 (75%)	39 (74%)	41 (76%)	146 (74%)	150 (75%)
	Female	38 (26%)	36 (25%)	14 (25%)	13 (24%)	52 (26%)	49 (25%)
ECOG							
	0	79 (54%)	96 (66%)	28 (53%)	28 (52%)	107 (54%)	124 (62%)
	1	58 (40%)	42 (29%)	22 (42%)	22 (41%)	80 (40%)	64 (32%)
	2	8 (6%)	7 (5%)	3 (6%)	4 (7%)	11 (6%)	11 (6%)
Ann–Arbor disease staging							
	II	7 (5%)	12 (8%)	2 (4%)	2 (4%)	9 (5%)	14 (7%)
	III	4 (3%)	5 (3%)	2 (4%)	5 (9%)	6 (3%)	10 (5%)
	IV	134 (92%)	128 (88%)	49 (92%)	47 (87%)	183 (92%)	175 (88%)
Blastoid disease							
	Present	12/142 (8%)	5/127 (4%)	3/50 (6%)	5/51 (10%)	15/192 (8%)	10/178 (6%)
	Missing	3	18	3	3	6	21
Ki67 ≥30%							
	Ki67 ≥30%	54/122 (44%)	37/103 (36%)	17/35 (49%)	18/39 (46%)	71/157 (45%)	55/142 (39%)
	Missing	23	42	18	15	41	57
MIPI risk category							
	Low	17/143 (12%)	17/144 (12%)	6/52 (12%)	6/54 (11%)	23/195 (12%)	23/198 (12%)
	Intermediate	42/143 (29%)	45/144 (31%)	19/52 (37%)	19/54 (35%)	61/195 (31%)	64/198 (32%)
	High	84/143 (59%)	82/144 (57%)	27/52 (52%)	29/54 (54%)	111/195 (57%)	111/198 (56%)
	Missing	2	1	1	0	3	1
*TP53* mutation							
	With mutation	14/54 (26%)	15/58 (26%)	4/21 (19%)	7/22 (32%)	18/75 (24·0%)	22/80 (28%)
	Missing	91	87	32	32	123	119
Country							
	Nordic	65 (45%)	62 (43%)	3 (6%)	5 (9%)	68 (34%)	67 (34%)
	UK	80 (55%)	83 (57%)	50 (94%)	49 (91%)	130 (66%)	132 (66%)

Data are n (%) unless otherwise stated. Ethnicity was not captured in the ENRICH trial. ECOG=Eastern Cooperative Oncology Group. MIPI=Mantle Cell Lymphoma International Prognostic Index. R-CHOP=rituximab–cyclophosphamide, doxorubicin, vincristine, and prednisolone.

94 (47%) of the 199 patients in the intervention group and 121 (61%) of 198 in the control group had disease progression or death before data cutoff. The median progression-free survival in the intervention group was 65·3 months (95% CI 52·7–not evaluable [NE]), compared with 42·4 months (95% CI 32·7–55·3) in the immunochemotherapy group.

**Figure 2:**
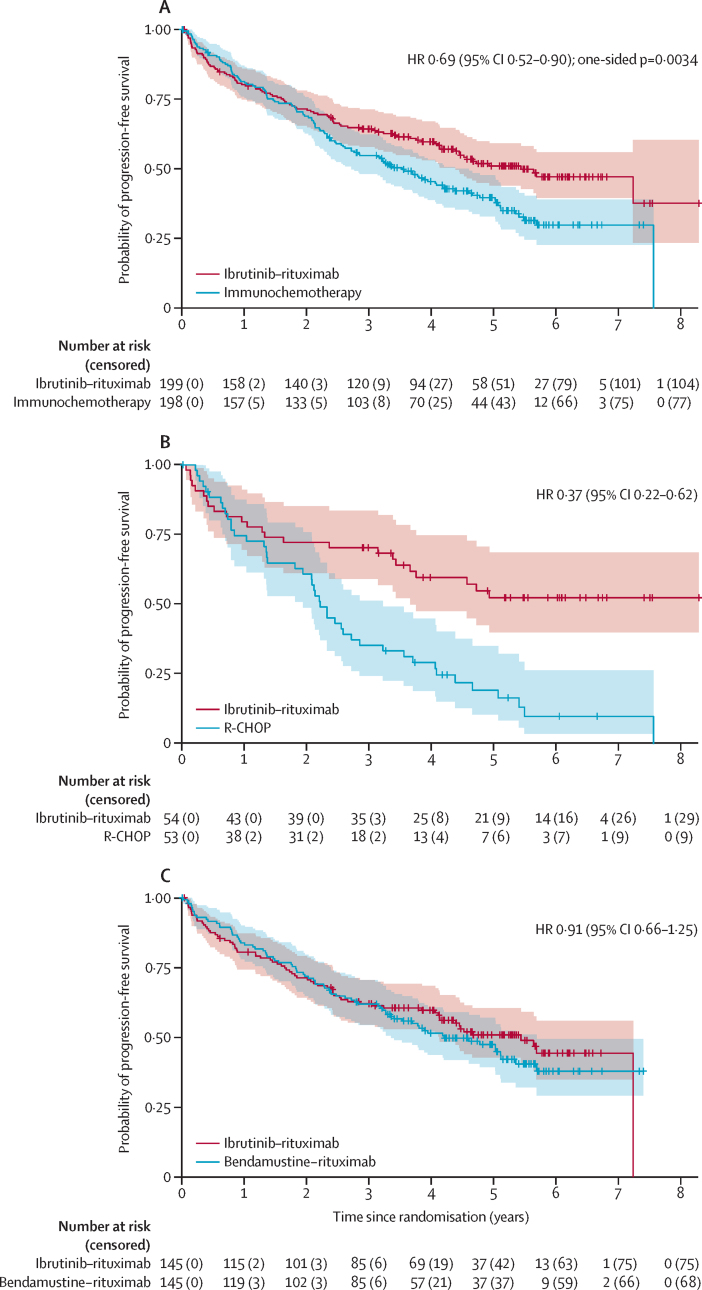
Progression-free survival Kaplan–Meier curves for all patients (A), the R-CHOP choice group (B), and the bendamustine–rituximab choice group (C) Progression-free survival Kaplan–Meier curves for the intention-to-treat population. (A) Overall Kaplan–Meier curve for ibrutinib–rituximab versus immunochemotherapy. (B) Pre-randomisation investigator choice of R-CHOP (ibrutinib–rituximab *vs* R-CHOP). (C) Pre-randomisation investigator choice of bendamustine–rituximab (ibrutinib–rituximab *vs* bendamustine–rituximab). HR=hazard ratio. R-CHOP=rituximab–cyclophosphamide, doxorubicin, vincristine, and prednisolone.

The primary analysis of progression-free survival showed superiority of ibrutinib–rituximab over immunochemotherapy (HR 0·69 [95% CI 0·52–0·90]; p=0·0034; figure 2; Kaplan–Meier curves for all participants and proportionality of hazards are shown in the appendix [pp 4–6]). Outcomes for ibrutinib–rituximab were similar in both randomised groups regardless of pre-randomisation choice of immunochemotherapy or induction cycle number received. The rituximab–bendamustine choice control group was substantially different from the R-CHOP choice control group, and prespecified testing showed a significant interaction between the choice of immunochemotherapy and treatment allocation (p=0·0038; appendix p 7). We evaluated the progression-free survival of ibrutinib–rituximab versus immunochemotherapy based on pre-randomisation choice of immunochemotherapy (figure 2, appendix p 8). For the R-CHOP subgroup, the HR for ibrutinib–rituximab compared to R-CHOP was 0·37 (95% CI 0·22–0·62). The 5-year progression-free survival probability was 52% (95% CI 40–69) for the ibrutinib–rituximab group compared with 19% (11–35) for the R-CHOP group. For the rituximab–bendamustine subgroup, the HR for ibrutinib–rituximab compared to rituximab–bendamustine was 0·91 (95% CI 0·66–1·25), with a 5-year progression-free survival probability of 51% (95% CI 43–60) for the ibrutinib–rituximab group and 47% (39–57) for the rituximab–bendamustine group. In an exploratory analysis, there were no substantial changes to the conclusions when country (UK *vs* Nordic) was added as a covariate to the primary analysis. Additional analyses performed for the per-protocol population for progression-free survival and for failure-free survival did not alter the conclusions of the primary analysis (appendix p 29).

**Figure 3:**
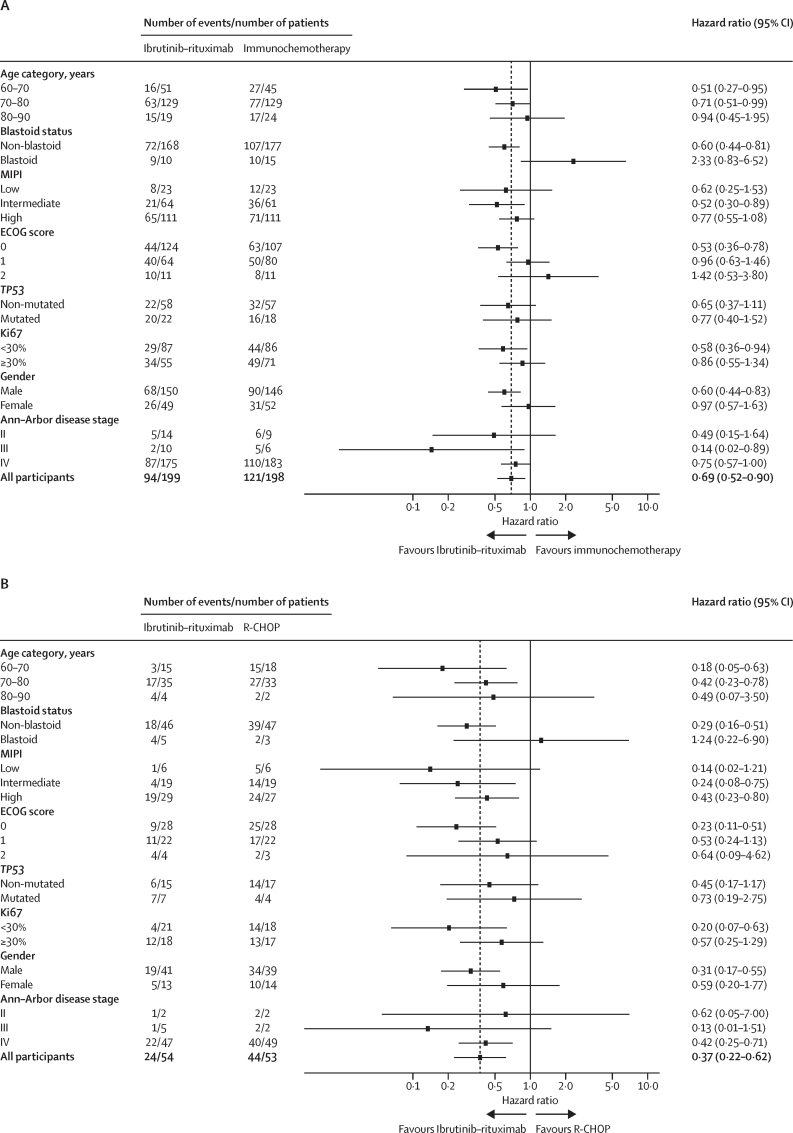

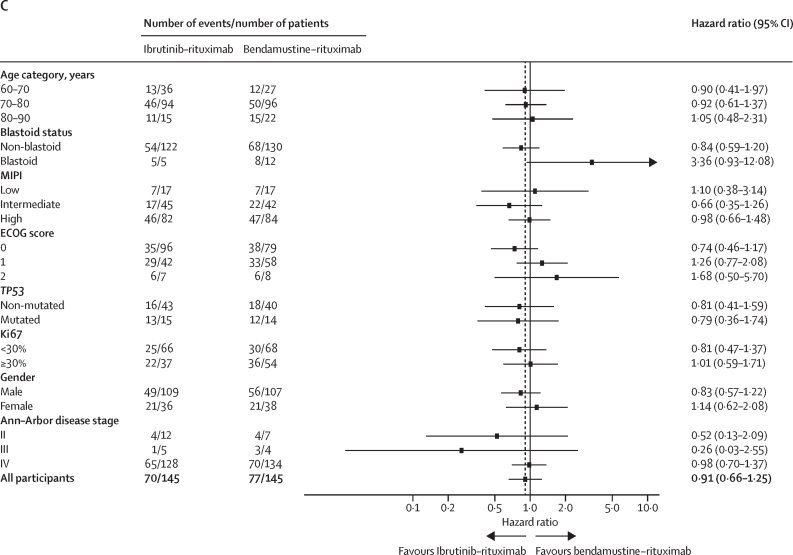
Subgroup analysis of progression-free survival Subgroup analysis of progression-free survival by treatment allocation, adjusted hazard ratios for ibrutinib–rituximab or immunochemotherapy. (A) Ibrutinib–rituximab versus immunochemotherapy where all comparisons are adjusted for the pre-randomisation investigator choice of immunochemotherapy. (B) Pre-randomisation investigator choice of R-CHOP (ibrutinib–rituximab *vs* R-CHOP). (C) Pre-randomisation investigator choice of bendamustine–rituximab (ibrutinib–rituximab *vs* bendamustine-rituximab). Analysis of subgroups MIPI and Ki67 with multiple imputation for missing grouping values are provided in the appendix (p 42). ECOG=Eastern Cooperative Oncology Group. MIPI=Mantle Cell Lymphoma International Prognostic Index. R-CHOP=rituximab–cyclophosphamide, doxorubicin, vincristine, and prednisolone.

The progression-free survival treatment benefit compared with immunotherapy was consistent across most predefined subgroups, with the exception of blastoid disease where a trend in favour of immunochemotherapy was seen (HR 2·33 [95% CI 0·83–6·52], median progression-free survival 6·9 months [95% CI 1·9–NE] for ibrutinib–rituximab *vs* 21·1 months [95% CI 9·8–NE] for immunochemotherapy; figure 3).

The treatment effect appeared greater in those with Ki67 under 30%, with a HR for progression-free survival of 0·58 (95% CI 0·36–0·94) for those treated with ibrutinib–rituximab compared with immunochemotherapy. The HR for those with Ki67 30% or greater was 0·86 (95% CI 0·55–1·34). 40 (26%) of 155 patients tested by next generation sequencing on baseline tumour samples found a pathogenic *TP53* mutation (22 [28%] of 80 for the intervention group *vs* 18 [24%] of 75 for the control group), including five of seven tumours with blastoid disease (appendix pp 27–28). For patients who had a *TP53* mutation, the median progression-free survival for those treated with ibrutinib–rituximab was 18·5 months (95% CI 4·2–46·2), versus 8·9 months (2·9–25·7) for those treated with immunochemotherapy (HR 0·77 [95% CI 0·40–1·52]; appendix pp 10–12; for progression-free survival by blastoid status and Ki67 percentage see the appendix pp 12–17).

Disease progression accounted for 49 (52%) of 94 progression-free survival events in the intervention group and 77 (64%) of 121 of progression-free survival events in the control group (including 33 [75%] of 44 for R-CHOP and 44 [57%] of 77 for rituximab–bendamustine). For the 145 patients treated with rituximab–bendamustine, six (4%) patients had disease progression during induction, 18 (12%) during maintenance, and 11 (8%) during follow-up, with nine (6%) progression events after cessation of treatment. For the 53 patients allocated to R-CHOP, three (6%), 18 (34%), and seven (13%) progression events occurred during the induction, maintenance, and follow-up periods, respectively, with five (9%) occurring following the cessation of treatment. For the 199 patients receiving ibrutinib–rituximab, 20 (10%), 17 (9%), and five (3%) progression-free survival events occurred during induction, maintenance and follow-up, respectively, with seven events (4%) following cessation of treatment (appendix p 30).

**Figure 4:**
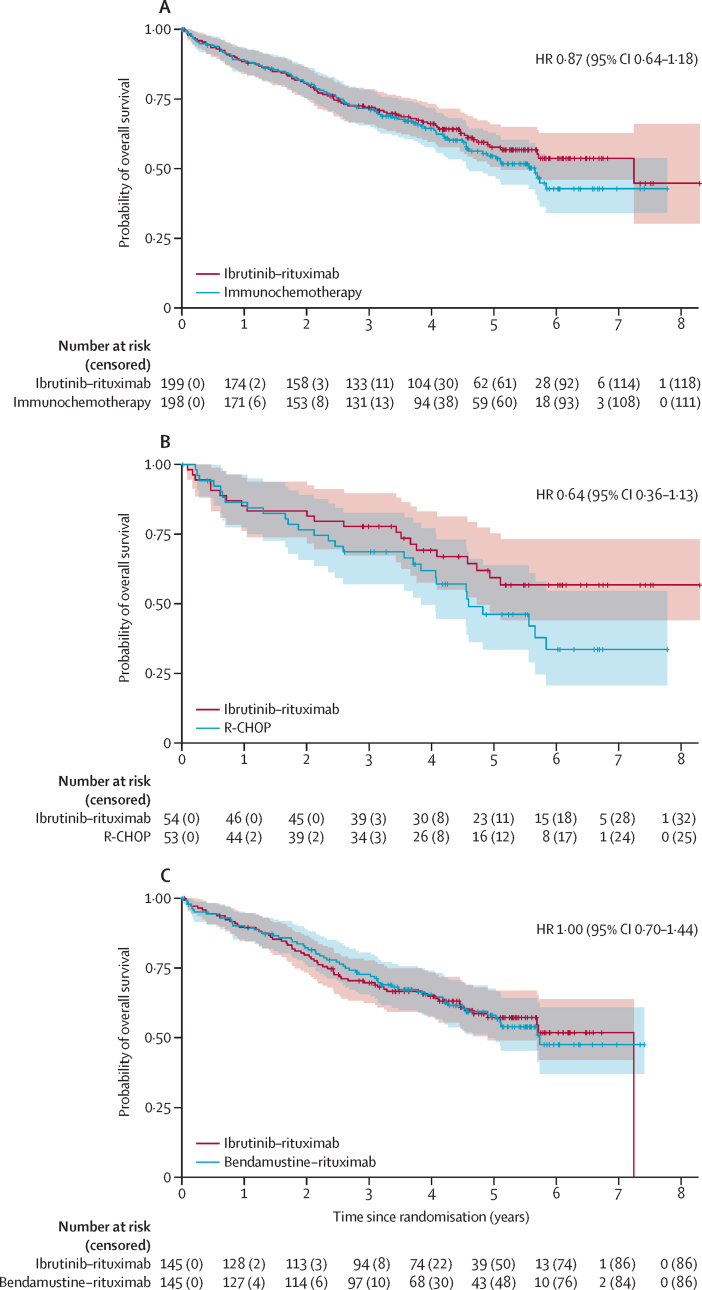
Overall survival Kaplan–Meier curves for all patients (A), the R-CHOP choice group (B) and the bendamustine–rituximab choice group (C) Overall survival Kaplan–Meier curves of the intention-to-treat population. (A) Overall Kaplan–Meier curve for ibrutinib–rituximab versus immunochemotherapy. (B) Pre-randomisation investigator choice of R-CHOP (ibrutinib–rituximab *vs* R-CHOP). (C) Pre-randomisation investigator choice of rituximab plus bendamustine (ibrutinib–rituximab *vs* bendamustine–rituximab). R-CHOP=rituximab–cyclophosphamide, doxorubicin, vincristine, and prednisolone.

The most common causes of non-relapse mortality were infection (23 [51%] of 45 events in the intervention group, including 14 associated with COVID-19, and 18 [41%] of 44 events in the control group, including nine associated with COVID-19) and cardiac causes (nine [20%] of 45 events in the intervention group *vs* eight [18%] of 44 events in the immunochemotherapy group). Causes and timing of non-relapse mortality are described in the appendix (p 32). At the cutoff date for the primary analysis, 80 deaths (40%) were observed in the intervention group and 87 deaths (44%) in the control group (including 28 [53%] of the 53 participants in the R-CHOP group and 59 [41%] of the 145 participants in the rituximab–bendamustine group). The HR for death comparing the intervention and the immunochemotherapy group was 0·87 (95% CI 0·64–1·18; figure 4, appendix p 18).

Mantle-cell lymphoma deaths accounted for 29 deaths (15%) in the ibrutinib–rituximab group versus 38 deaths (19%) in the immunochemotherapy group. COVID-19 was associated with 19 deaths in the ibrutinib–rituximab group versus 14 in the immunochemotherapy group. Infection (excluding COVID-19) caused 11 deaths (6%) in the ibrutinib–rituximab group (of which two occurred in induction, four in maintenance, three in follow-up post-maintenance, and two after cessation of treatment) compared with 11 deaths (6%) in the immunochemotherapy group (of which four occurred in induction, three in maintenance, one in follow-up post-maintenance, and three after treatment cessation). Cardiac deaths accounted for ten deaths (5%) in the ibrutinib–rituximab group compared with ten (5%) in the immunochemotherapy group. Causes of death by pre-randomisation choice of chemotherapy and group are shown in the appendix (pp 19–21, 33). Sudden deaths were reported in 18 participants in the intervention group and 13 in the immunochemotherapy group, of which eight (44%) and six (46%) deaths, respectively, were thought to be cardiac-related (appendix p 34). Not all deaths in the trial were associated with, or reported as, adverse events, so treatment-relatedness cannot always be determined. Timings of deaths related to time on treatment are shown in the appendix (p 35).

The overall response rate was 171 (86%) of the 199 patients who underwent random allocation for ibrutinib–rituximab versus 169 (85%) of 198 for immunochemotherapy (47 [89%] of 53 for R-CHOP and 122 [84%] of 145 for rituximab–bendamustine). A complete response was observed in 107 (54%) of 199 patients for ibrutinib–rituximab, and in 105 (53%) of 198 for immunochemotherapy (25 [47%] of 53 for R-CHOP and 80 [55%] of 145 for rituximab–bendamustine; appendix p 31).

The 5-year overall survival probability was 58% (95% CI 51–66) for the intervention group versus 55% (47–63) for the control group (HR 0·87 [95% CI 0·64–1·18]; figure 4). For those with a pre-randomisation choice of R-CHOP, the 5-year survival probability for the ibrutinib–rituximab group was of 59% (95% CI 47–75) compared to 46% (34–64) for the R-CHOP group (HR 0·64 [95% CI 0·36–1·13]). For the pre-randomisation investigator choice of rituximab–bendamustine, the 5-year survival probability was 57% (95% CI 49–67) for the ibrutinib–rituximab group and 58% (50–68) for the rituximab–bendamustine group (HR 1·00 [95% CI 0·70–1·44]).

Results from the analysis of time-to-nex-treatment did not substantially change conclusions from the primary analysis of progression-free survival (appendix p 30). Second-line therapy was received by 42 (21%) of 199 patients in the intervention group compared with 69 (35%) of 198 patients in the immunochemotherapy group. The most common second-line treatment for the intervention group was R-bendamustine (15 [36%] of 42), followed by R-CHOP (eight [19%] of 42) and R-BAC (rituximab, bendamustine, and cytarabine; eight [19%] of 42; appendix p 36). The most common second-line therapy following immunochemotherapy was a covalent BTK inhibitor (46 [67%] of 69 of patients receiving a follow-up therapy; appendix p 37).

Health-related quality of life improved more rapidly in the ibrutinib–rituximab group compared to immunochemotherapy. For ibrutinib–rituximab, the median EORTC QLQ-C30 score rose from 86 (IQR 69–94) to 91 (84-95) at the mid-induction timepoint, compared with 85 (IQR 73–93) to 85 (76–92) for the immunochemotherapy group. EORTC QLQ-C30 scores were similar between the ibrutinib–rituximab group and immunochemotherapy groups at the end of maintenance (appendix pp 22, 37).

During induction and maintenance, in patients who received at least one round of treatment, 99 (69%) of 143 receiving bendamustine–rituximab, 37 (71%) of 52 receiving R-CHOP, and 132 (67%) of 198 receiving ibrutinib–rituximab reported at least one adverse event of grade 3 or above (appendix pp 38–39). Data for the full adverse event reporting period are given in the appendix (p 39). Patients could have more than one grade 3 event at a time; grade 3 or above haematological adverse events were reported in 33 (17%) of 198, 26 (50%) of 52, and 48 (34%) of 143 in the intervention, R-CHOP, and rituximab–bendamustine groups, respectively. Grade 3 or above non-haematological adverse events were reported in 120 (61%) of 198, 27 (52%) of 52, and 75 (52%) of 143 of the intervention, R-CHOP, and rituximab–bendamustine groups, respectively (table 2).

**Table 2:** Adverse events of grade 3 and above during induction and maintenance treatment

		**Induction treatment**						**Maintenance treatment**					
		Bendamustine–rituximab (n=143)*		R-CHOP (n=52)†		Ibrutinib–rituximab (n=198)‡		Bendamustine–rituximab (n=143)*		R-CHOP (n=52)†		Ibrutinib–rituximab (n=198)‡	
		Number of patients with grade ≥3 event (% of safety population)	Events per participant-year	Number of patients with grade ≥3 event (% of safety population)	Events per participant-year	Number of patients with grade ≥3 event (% of safety population)	Events per participant-year	Number of patients with grade ≥3 event (% of safety population)	Events per participant-year	Number of patients with grade ≥3 event (% of safety population)	Events per participant-year	Number of patients with grade ≥3 event (% of safety population)	Events per participant-year
Total adverse events, n_patients_ (% of safety population)		73 (51%)	2·287	35 (67%)	3·178	83 (42%)	1·800	44 (31%)	0·475	11 (21%)	0·297	97 (49%)	0·676
All cardiac events^§^		7 (5%)	0·170	5 (10%)	0·218	22 (11%)	0·351	2 (1%)	0·011	3 (6%)	0·078	29 (15%)	0·149
All bleeding events¶		2 (1%)	0·046	3 (6%)	0·174	6 (3%)	0·099	1 (1%)	0·006	0	0	5 (3%)	0·019
Blood and lymphatic system disorders		29 (20%)	0·726	16 (31%)	1·175	19 (10%)	0·340	12 (8%)	0·11	3 (6%)	0·063	18 (9%)	0·100
	Anaemia	3 (2%)	0·062	3 (6%)	0·174	4 (2%)	0·055	..	..	..	..	..	..
	Febrile neutropenia	6 (4%)	0·108	5 (10%)	0·261	3 (2%)	0·033	..	..	..	..	..	..
	Neutropenia	19 (13%)	0·448	9 (17%)	0·479	8 (4%)	0·132	10 (7%)	0·094	3 (6%)	0·063	14 (7%)	0·078
	Thrombocytopenia	7 (5%)	0·108	2 (4%)	0·261	9 (5%)	0·099	..	..	..	..	..	..
Cardiac disorders		4 (3%)	0·093	2 (4%)	0·087	11 (6%)	0·154	1 (1%)	0·006	2 (4%)	0·047	14 (7%)	0·067
	Atrial fibrillation	1 (1%)	0·031	0	0	5 (3%)	0·055	0	0	0	0	9 (5%)	0·037
Gastrointestinal disorders		10 (7%)	0·185	4 (8%)	0·174	5 (3%)	0·110	1 (1%)	0·006	0	0	6 (3%)	0·026
	Diarrhoea	4 (3%)	0·062	0	0	1 (1%)	0·022	..	..	..	..	..	..
General disorders and administration site conditions		9 (6%)	0·185	2 (4%)	0·087	8 (4%)	0·110	..	..	..	..	..	..
	Pyrexia	6 (4%)	0·093	1 (2%)	0·044	5 (3%)	0·066	..	..	..	..	..	..
Infections and infestations		16 (11%)	0·371	12 (23%)	0·914	23 (12%)	0·307	21 (15%)	0·177	3 (6%)	0·063	36 (18%)	0·197
	Infection	4 (3%)	0·077	0	0	1 (1%)	0·011	..	..	..	..	..	..
	Neutropenic sepsis	2 (1%)	0·031	8 (15%)	0·522	4 (2%)	0·066	..	..	..	..	..	..
	Pneumonia	3 (2%)	0·077	2 (4%)	0·087	3 (2%)	0·033	5 (3%)	0·028	1 (2%)	0·016	8 (4%)	0·041
	Sepsis	3 (2%)	0·046	3 (6%)	0·131	3 (2%)	0·033	0	0	0	0	5 (3%)	0·019
	Coronavirus infection‖	..	..	..	..	..	..	10 (7%)	0·094	1 (2%)	0·016	14 (7%)	0·059
Injury, poisoning, and procedural complications		4 (3%)	0·062	4 (8%)	0·261	2 (1%)	0·022	7 (5%)	0·039	1 (2%)	0·016	2 (1%)	0·007
	Infusion related reaction	2 (1%)	0·031	3 (6%)	0·174	1 (1%)	0·011	..	..	..	..	..	..
Investigations		8 (6%)	0·170	4 (8%)	0·174	5 (3%)	0·055	7 (5%)	0·039	2 (4%)	0·031	4 (2%)	0·015
	Lymphocyte count decreased	4 (3%)	0·108	1 (2%)	0·044	0	0	..	..	..	..	..	..
Metabolism and nutrition disorders		7 (5%)	0·108	1 (2%)	0·044	8 (4%)	0·099	3 (2%)	0·028	0	0	5 (3%)	0·03
Musculoskeletal and connective tissue disorders		0	0	0	0	5 (3%)	0·077	..	..	..	..	..	..
Neoplasms benign, malignant, and unspecified (including cysts and polyps)		..	..	..	..	..	..	6 (4%)	0·033	1 (2%)	0·016	10 (5%)	0·037
Nervous system disorders		2 (1%)	0·031	3 (6%)	0·174	2 (1%)	0·022	..	..	..	..	..	..
Respiratory, thoracic, and mediastinal disorders		6 (4%)	0·124	1 (2%)	0·044	9 (5%)	0·11	1 (1%)	0·006	1 (2%)	0·016	7 (4%)	0·03
Skin and subcutaneous tissue disorders		9 (6%)	0·139	0	0	6 (3%)	0·066	..	..	..	..	..	..
	Rash	5 (3%)	0·077	0	0	2 (1%)	0·022	..	..	..	..	..	..
Vascular disorders		3 (2%)	0·046	1 (2%)	0·044	13 (7%)	0·22	1 (1%)	0·006	1 (2%)	0·016	12 (6%)	0·059
	Hypertension	1 (1%)	0·015	1 (2%)	0·044	10 (5%)	0·187	1 (1%)	0·006	1 (2%)	0·016	12 (6%)	0·059

Data are number of patients with at least one grade 3 event (percentage of safety population). Adverse events are reported by MedDRA preferred term and organ system class, presented if they occurred in at least 3% of the safety population within any treatment group. The safety population includes all participants who completed at least one cycle of induction treatment. Adverse events were classified as haematological if the MedDRA preferred term included thrombocytopenia, neutropenia, anaemia, febrile neutropenia, platelet count decreased, white blood cell count decreased, neutropenic infection, neutropenic sepsis, neutrophil count decreased, haemoglobin decreased, lymphocyte count decreased, or lymphopenia. For the 143 patients in the safety population who received bendamustine–rituximab, 86 (60%) had a grade 3 events, 23 (16%) had grade 4 events, and six (4%) had grade 5 events. For the 52 patients allocated to R-CHOP, 31 (60%) had grade 3 events; 11 (21%) had grade 4 events, and four (8%) had grade 5 events. For the 198 patients assigned to ibrutinib–rituximab, 117 (59%) had grade 3 events, 30 (15%) had grade 4 events, and 15 (8%) had grade 5 events.

* Patients allocated to bendamustine–rituximab were exposed for 64·7 participant-years in the induction phase and 181·0 participant-years in the maintenance phase.

† Patients allocated to R-CHOP were exposed for 23·0 participant-years in the induction phase and 63·9 participant-years in the maintenance phase.

‡ Patients in the intervention group were exposed for 91·1 participant-years in the induction phase and 269·2 participant-years in the maintenance phase.

¶ All adverse events classified as bleeding-related.

‖ All grade 3 and above coronavirus infections reported refer to COVID-19. R-CHOP=rituximab–cyclophosphamide, doxorubicin, vincristine, and prednisolone.

Of the grade 3 or above haematological adverse events in the induction phase, neutropenia occurred in 18 (9%) of 198 for ibrutinib–rituximab, 11 (21%) of 52 for R-CHOP, and 27 (19%) of 143 for rituximab–bendamustine. Grade 3 or above hypertension was reported in 22 (11%) of 198 for ibrutinib–rituximab, two (4%) of 52 for R-CHOP, and two (1%) of 143 for rituximab–bendamustine, respectively. Atrial fibrillation was reported in 13 (7%) of 198 of the ibrutinib–rituximab group compared with one (1%) of 195 in the immunochemotherapy group. 11 (6%) of 198 patients in the ibrutinib–rituximab group had bleeding-related adverse events compared with six (3%) of 195 in the immunochemotherapy group. During induction treatment, 83 (42%) of 198 patients on ibrutinib–rituximab, 35 (67%) of 52 patients on R-CHOP, and 73 (51%) of 143 patients on rituximab–bendamustine had grade 3 or above adverse events (table 2). Grade 3 or above infection-related adverse events were seen in 23 (12%) of 198, 12 (23%) of 52, and 16 (11%) of 143 patients treated with ibrutinib–rituximab, R-CHOP, and rituximab–bendamustine, respectively. In the maintenance phase, 97 (49%) of 198 patients, 11 (21%) of 52 patients, and 44 (31%) of 143 patients in the ibrutinib–rituximab, R-CHOP and rituximab–bendamustine groups, respectively (of these, 29 [15%] of 198 patients, three [6%] of 52 patients, and two [1%] of 143 patients, respectively, had cardiac events) had adverse events of grade 3 or above. Infection-related adverse events of grade 3 or above were observed in 36 (18%) of 198 patients, three (6%) of 52 patients, and 21 (15%) of 143 patients, respectively.

The median time on ibrutinib–rituximab was 33·8 months (95% CI 27·8–43·0; data not shown) and 104 (52%) of the 199 patients allocated to ibrutinib–rituximab discontinued treatment while continuing trial follow-up. The most common reasons for stopping treatment included disease progression (41 [21%] of 199 patients), cardiac reasons (15 [8%] patients), infection (nine [5%] patients), respiratory (6 [3%] of patients), and second malignancy (5 [3%] of patients). Of infectious causes, three were COVID-19-related, and other infections included three respiratory infections, two unknown, and one urological. Further reasons for discontinuation can be seen by pre-randomisation investigator choice of treatment and allocated group in the appendix (pp 40–41).

Of those randomly allocated to ibrutinib–rituximab, 169 (85%) of 199 completed induction and 105 (53%) of 199 completed maintenance rituximab. For those randomly allocated to immunochemotherapy, 110 (76%) of 145 and 42 (79%) of 53 patients assigned to rituximab–bendamustine and R-CHOP, respectively, completed induction. 66 (46%) of 145 and 18 (34%) of 53 of patients randomised to rituximab–bendamustine and R-CHOP completed maintenance, respectively. Three patients receiving R-CHOP and four receiving rituximab–bendamustine had an assessment of stable disease at mid-induction, with two participants in each group stopping treatment at this timepoint (appendix p 32). No patients on ibrutinib–rituximab stopped treatment with stable disease (figure 1).

Recruitment and follow-up were conducted during the COVID-19 pandemic, with COVID-19 associated with 19 deaths in the intervention group and 14 deaths in the control group (two deaths in the R-CHOP group and 12 in the rituximab–bendamustine group). 26 (79%) of the 33 deaths occurred in patients before progression and contributed to 16 (17%) of 94 progression-free survival events in the intervention group and ten (8%) of 121 in the control group (two [5%] of 44 for R-CHOP and eight [10%] of 77 for rituximab–bendamustine). In the control group, no patients died of COVID-19 during induction, four died during maintenance, and six died post-maintenance, compared with one, six, and ten deaths in the ibrutinib–rituximab group, respectively. Most deaths (31 [94%] of 33) occurred following the introduction of routine vaccination in each recruiting country.

A progression-free survival sensitivity analysis censoring at the last visit before COVID-19 death did not alter the conclusions from the primary analysis (see appendix pp 24–27, 30).

## Discussion

To our knowledge, this study, which randomly allocated patients to treatment with ibrutinib–rituximab or to standard immunochemotherapy, is the first randomised phase 3 study to demonstrate a significant improvement in progression-free survival in previously untreated mantle cell lymphoma. According to a predefined statistical plan, this was primarily driven by worse outcomes in the R-CHOP control group compared to the ibrutinib–rituximab intervention group, whereas the progression-free survival for ibrutinib–rituximab and rituximab–bendamustine were broadly comparable.

Additionally, ibrutinib–rituximab was associated with a faster improvement in health-related quality of life compared with either of the immunochemotherapy options at the mid-induction treatment timepoint, with reported toxicities in keeping with the known safety profile. Ibrutinib–rituximab was associated with less haematological toxicity compared with immunochemotherapy, but more non-haematological toxicity. Furthermore, more patients successfully completed induction treatment in the ibrutinib–rituximab group, despite having more disease progression events during induction compared with immunochemotherapy. In keeping with the known adverse event profile of ibrutinib and the continuous drug exposure, 7% of patients in the ibrutinib–rituximab group had grade 3 or above atrial fibrillation. Notably, patients treated with ibrutinib–rituximab had a higher frequency of adverse events during the maintenance phase of the study, and cardiac adverse events were reported by 15% of patients treated with ibrutinib–rituximab patients compared to 3% of patients treated with immunochemotherapy during maintenance. However, more patients randomly allocated to ibrutinib–rituximab completed maintenance. Overall, there were similar numbers of progression-free deaths in the ibrutinib–rituximab group and the immunochemotherapy groups, regardless of choice of chemotherapy. The number of deaths thought to be cardiac-related was similar in the ibrutinib–rituximab group and the control group. Infections were a major cause of death over the entire trial up until end of safety monitoring, causing 28 deaths (14% of patients) in the ibrutinib–rituximab group. By contrast, in the SHINE trial, which recruited patients from May 2013 to November 2014, 12 (5%) of 261 patients died of infection.^17^ This difference relates to the large number of patients that died of COVID-19 in ENRICH, which accounted for 28 (56%) of 50 infectious deaths.

The overall survival was similar across the whole trial but was numerically improved with ibrutinib–rituximab in those with a pre-randomisation choice of R-CHOP. Investigators were able to choose which immunochemotherapy to use for individual patients, and patients' baseline characteristics were similar between those who received R-CHOP and rituximab–bendamustine. Although the trial was not specifically designed to directly compare R-CHOP with rituximab–bendamustine, and caution is needed when interpreting these data, survival outcomes for patients treated with rituximab–bendamustine were numerically superior when indirectly compared to those treated with R-CHOP.

The outcomes in the R-CHOP subgroup were worse than some reported data, despite the use of rituximab maintenance.^4^ This was primarily due to an excess of progression events in the R-CHOP group, of which the majority occurred during the maintenance phase: there were 18 disease progression events during the maintenance phase compared with three in those with pre-randomisation choice R-CHOP randomised to ibrutinib–rituximab. There is a wide variation in reported outcomes from R-CHOP in mantle-cell lymphoma, which might reflect different populations and maintenance strategies studied.^2^, ^4^

Features including *TP53* mutation, high proliferation rate as measured by the expression of Ki67, and blastoid disease are associated with poor outcomes with standard chemotherapy.^21^, ^22^, ^23^, ^24^ Interoperator variation in Ki67 expression is well described.^25^ Despite no central laboratory reporting, blastoid disease and Ki67 expression remained strong prognostic factors in this study. 26% of tested tumours had a *TP53* mutation, including five of seven tumours with blastoid disease. While interpretation should be treated with caution owing to relatively small numbers, the progression-free survival within the *TP53* mutated group was numerically prolonged in those treated with ibrutinib–rituximab compared with immunochemotherapy. In this trial, the median progression-free survival for patients treated with ibrutinib–rituximab who had *TP53* mutations was 18·5 months. In comparison, progression-free survival for 26 patients with a *TP53* mutation treated with R-bendamustine plus ibrutinib in the SHINE study was 28·8 months, and the SYMPATICO study included 29 patients with *TP53* mutations who were treated with first-line ibrutinib plus venetoclax with a median progression-free survival of 19·8 months months.^17^, ^26^ Patients with blastoid disease had longer progression-free survival when treated with immunochemotherapy, suggesting that ibrutinib–rituximab might be inadequate treatment for these patients, although as only 25 patients with blastoid disease were enrolled, this interpretation should be treated with caution. Ongoing trials are assessing the role of anti-CD20 antibody and BTK inhibitor combinations with or without BCL2 inhibitor therapy and may provide more information about the value of additional non-chemotherapeutic approaches to various subgroups.^27^, ^28^

This trial was substantially affected by the COVID-19 pandemic. There were more COVID-19 events in the intervention group than in the control group but censoring for these events did not alter conclusions either overall or by subgroup. The SHINE and ECHO trials both examined the addition of a BTK inhibitor to a rituximab–bendamustine backbone and reported a median progression-free survival of 80·6 months and 66·4 months respectively.^17^, ^29^ In the ENRICH trial, the progression-free survival of those patients treated with ibrutinib–rituximab when censoring for COVID-19 events was very similar to those treated with rituximab–bendamustine and ibrutinib in the SHINE trial, with similar performance of the rituximab–bendamustine groups in both studies. Furthermore, the COVID-19-censored progression-free survival was particularly good for those patients with Ki67 under 30% treated with ibrutinib–rituximab. This is consistent with other recent phase 2 data suggesting patients with low biological risk mantle-cell lymphoma are well served by the anti-CD20 and covalent BTK inhibitor doublet.^18^, ^30^

Risk stratification at diagnosis might aid future decision-making when selecting anti-CD20 and covalent BTK inhibitor doublet therapy in patients with untreated mantle-cell lymphoma. In deciding between different approaches, factors such as the proliferation index and morphological subtype of the tumour, the wish for time-limited versus ongoing treatment, and the different toxicity profiles should be considered. Furthermore, as T-cell-engaging treatments become more widely used in earlier lines of treatment there might be a preference to avoid bendamustine-containing regimens within the broader the treatment paradigm.

In conclusion, we report the results from the first known randomised trial to demonstrate superiority of the chemotherapy-free regimen ibrutinib–rituximab compared to immunochemotherapy in the first-line setting for older patients with mantle-cell lymphoma. Outcomes compared to the rituximab–bendamustine subgroup were broadly equivalent. Ibrutinib-rituximab should be considered a new standard of care treatment option for older patients unsuitable for intensive approaches.

### Contributors

### Data sharing

Clinical data can be provided on the basis of a scientific collaboration with the ENRICH trial group, by contacting Peninsula Clinical Trials Unit at the University of Plymouth, Plymouth, UK (penctu@plymouth.ac.uk).

## Declaration of interests

DJL reports consultancy and honoraria from AstraZeneca, Johnson and Johnson, Beigene, Roche, AbbVie, and Lilly. MJ reports honoraria from AbbVie, Johnson and Johnson, Roche, AstraZeneca, Kite (Gilead), and Lilly. IF reports consultancy fees from Kite (Gilead), Takeda, and Johnson and Johnson. CB reports consultancy and honoraria from Roche, Kite (Gilead), Takeda, Johnson and Johnson, AbbVie, and AstraZeneca. NC reports honoraria from AbbVie. MJB reports honoraria from Roche, Takeda, Celltrion, Kite (Gilead), Lilly, AbbVie, and Recordati; consulting or advisory roles for Incyte, Roche, Lilly, and AbbVie; and research funding, unrelated to the current work from Roche and Takeda. TAE reports consultancy and honoraria from Beigene, AstraZeneca, Roche, Gilead, Kite (Gilead), Takeda, Johnson and Johnson, Loxo Oncology, Incyte, Autolus, Nurix, and Galapagos. SR is a current employee of AstraZeneca. JR reports consultancy for Roche, AstraZeneca, and Kite (Gilead). AP reports consultancy or advisory roles for Beigene, AbbVie, Gilead, and Incyte. NM reports consultancy and honoraria from Takeda, Amgen, AbbVie, and Kite (Gilead). All other authors report no competing interests.
