# Supplementary material for: Ibrutinib and rituximab versus immunochemotherapy in patients with previously untreated mantle cell lymphoma (ENRICH): a randomised, open-label, phase 2/3 superiority trial
Source: Lancet. 2025 Oct 25;406(10514):1953–68. doi: 10.1016/S0140-6736(25)01432-1 (PMC12549478; doi:10.1016/S0140-6736(25)01432-1)
Supplement: Supplementary appendix [file mmc1.pdf]

THE LANCET

Supplementary appendix

This appendix formed part of the original submission and has been peer reviewed. We post it as supplied by the authors.

Supplement to: Lewis DJ, Jerkeman M, Sorrell L, et al. Ibrutinib and rituximab versus immunochemotherapy in patients with previously untreated mantle cell lymphoma (ENRICH): a randomised, open-label, phase 2/3 superiority trial. *Lancet* 2025; published online Oct 3. https://doi.org/10.1016/S0140-6736(25)01432-1.

Supplementary to: Lewis D et al. Ibrutinib and Rituximab versus Immunochemotherapy in previously untreated mantle cell lymphoma: a randomised open label phase II/III superiority trial.

Table of contents

Table of contents 1

Enrich trial investigators 2

Figure S1: Consort diagram 3

Figure S2: Kaplan-Meier curves for all participants 4

Figure S3: Assessment of the proportional hazards assumption for progression-free survival 5

Figure S4: Progression-free survival by immunochemotherapy and treatment allocation 7

Figure S5: Progression-free survival by investigator choice of immunochemotherapy 8

Figure S6: Kaplan-Meier plots of progression-free survival by TP53 mutation status 9

Figure S7: Kaplan-Meier plots of progression-free survival by blastoid status 12

Figure S8: Kaplan-Meier plots of progression-free survival by Ki67 status 15

Figure S9: Assessment of the proportional hazards assumption for overall survival 18

Figure S10: Causes of death by treatment allocation and pre-randomisation choice of immunochemotherapy 19

Figure S11: Quality of Life (EORTC QLQ-C30) scores by treatment taken (pre-randomisation investigator choice of immunochemotherapy and intervention) 22

Figure S12: COVID-19 censored Kaplan-Meier plots for progression-free survival 23

Figure S13: COVID-19 censored Kaplan-Meier plots for overall survival 25

Table S1 Baseline participant characteristics by pre-randomization investigator choice of immunochemotherapy and treatment allocation of the 25 participants with blastoid disease. 27

Table S2: Supplementary and sensitivity analyses 29

Table S3: Progressive disease timing 30

Table S4: Disease response by pre-randomisation choice of immunochemotherapy and treatment allocation. 31

Table S5: Causes of non-relapse mortality 32

Table S6: Cause of death by pre-randomisation choice of immunochemotherapy and treatment allocation. 33

Table S7: Cause of sudden death by pre-randomisation choice of immunochemotherapy and treatment allocation. 34

Table S8: Death timing 35

Table S9: Second line MCL treatment by pre-randomisation investigator choice of immunochemotherapy and treatment allocation 36

Table S10: Quality of Life (EORTC QLQ-C30) by pre-randomisation investigator choice of immunochemotherapy and treatment allocation 37

Table S11: Adverse events of grade 3 and above during induction treatment and maintenance. ...38

Table S12: Treatment discontinuation by pre-randomisation investigator choice of immunochemotherapy and treatment allocation 40

Missing data 42

Summary of relevant protocol deviations 43

Enrich trial investigators

Country (ethical approval number)	Principle Investigators
UK (15/WM/0268)	David Lewis (formerly Simon Rule), Mark Bishton, Toby Eyre, Chris McNamara, Nick Morley, Michelle Furtado (formerly Adam Forbes), Cathy Burton, Pam McKay, Nimish Shah, Andrew Davies, Meghna Ruparelia (formerly Jo Addada), Shankara Paneesha, Andrew Hodson, Deborah Turner, Fiona Miall, Marian Macheta, Renata Walewska, Clare Rowntree, Sunil Iyengar, Kim Linton, Rebecca Auer, Moya Young, Arvind Arumainathan, Santosh Narat, James Milnthorpe (formerly Jonathan Cullis), Iain Singer, Annika Whittle, Dominic Culligan, Rory McCulloch (formerly Richard Lush), Angus Broom, Mohsen Norouzi, Sudarshan Gurung (formerly Maadh Aldouri), Marketa Wilson, Piers Patten, Yasmin Hasan, Paul Kerr, Beth Harrison, Peter Forsyth, Moez Dungarwalla, Unmesh Mohite, Oonagh Sheehy, Wendy Osborne, Gavin Campbell, Russell Patmore, Vikram Singh, Fiona Cutler (formerly Paul Micallef-Eynaud), Rachel Boulton-Jones, Ruth Pettengell
Sweden (Dnr 2017/285)	Ingrid Glimelius, Mats Jerkeman, Kristina Sonnevi, Ingemar Lagerlöf, Lena Brandefors, Nevzeta Kuric, Catharina Lewerin, Karin Papworth
Norway (2017/1771/REK midt)	Jon Riise, Karin Wader
Denmark (S-20170123)	Jacob Haaber, Christian Poulsen, Hans Bentzen, Par Josefsson
Finland (HUS 1399 2019)	Annika Pasanen, Marjukka Pollari

Figure S1: Consort diagram

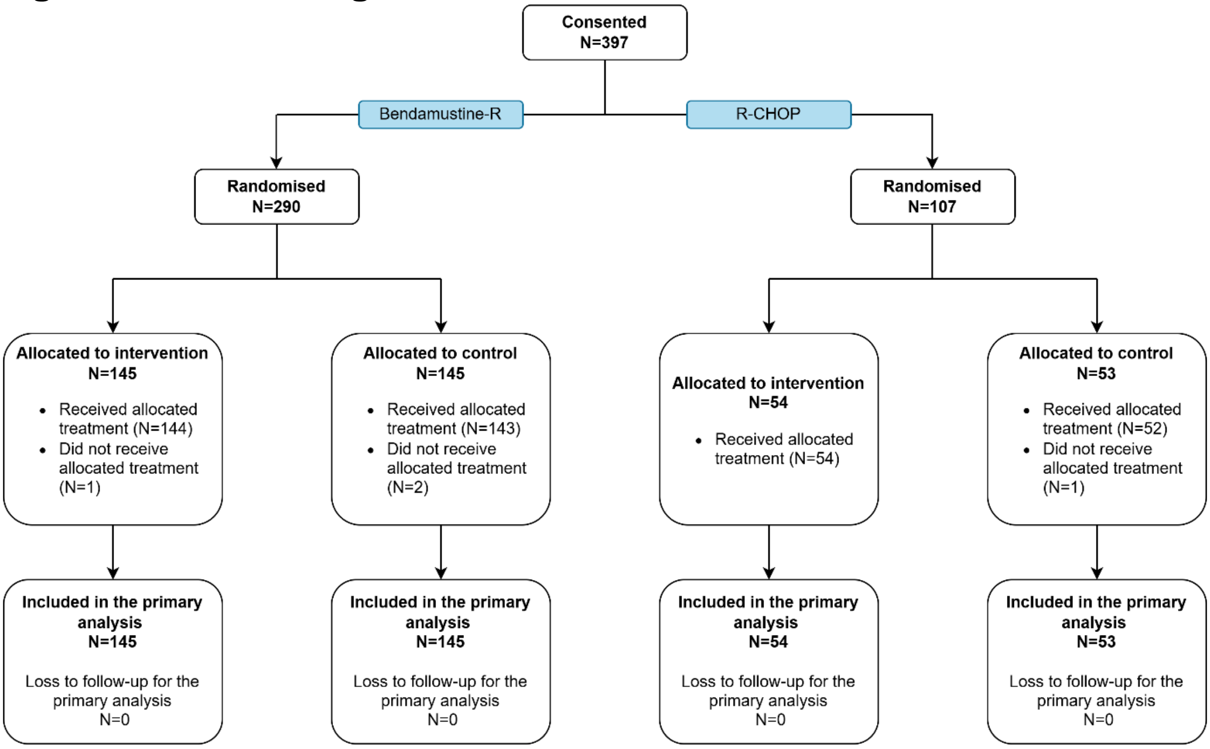

Participants who have withdrawn consent to follow-up without progressive disease were included in the primary analysis, censored at the last known progressive disease-free date. Reasons for withdrawal of consent are provided below.

- Intervention (choice = Bendamustine-R): N=10 (investigator decision: n=4, participant request: n=6)
- Control (choice = Bendamustine-R): N=10 (investigator decision: n=5, participant request: n=4, recorded progressive disease where participant transferred to hospice: n=1)
- Intervention (choice = R-CHOP): N=0
- Control (choice= R-CHOP): N=3 (ineligible: n=1, investigator decision: n=1, participant request: n=1)

Figure S2: Kaplan-Meier curves for all participants

Fig A – Progression-free survival. Fig B – Overall survival.

A

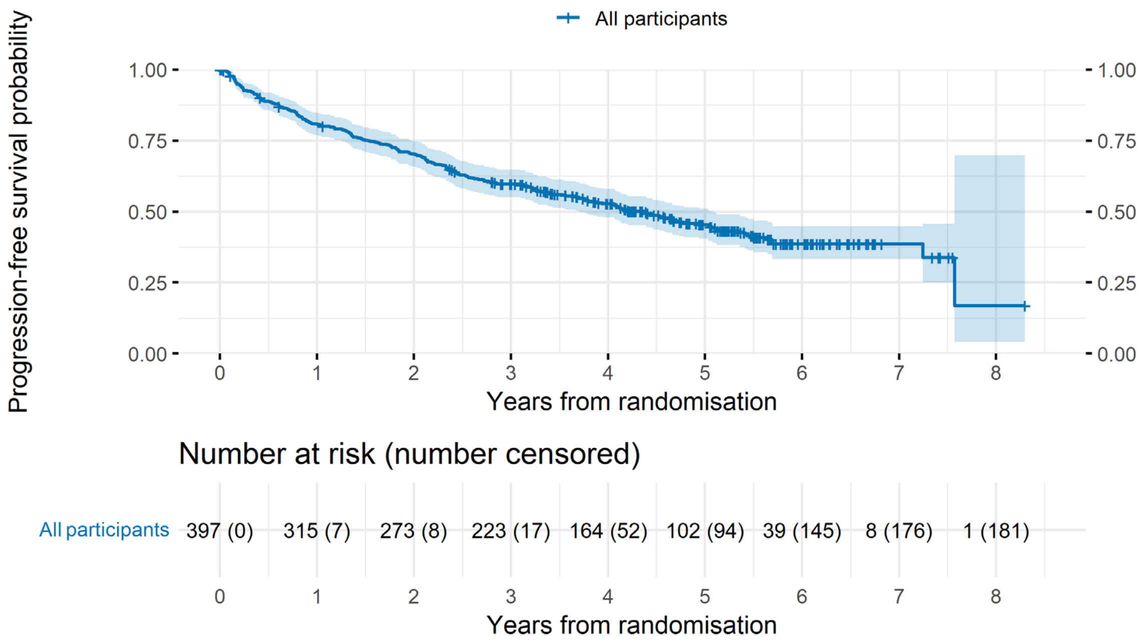

B

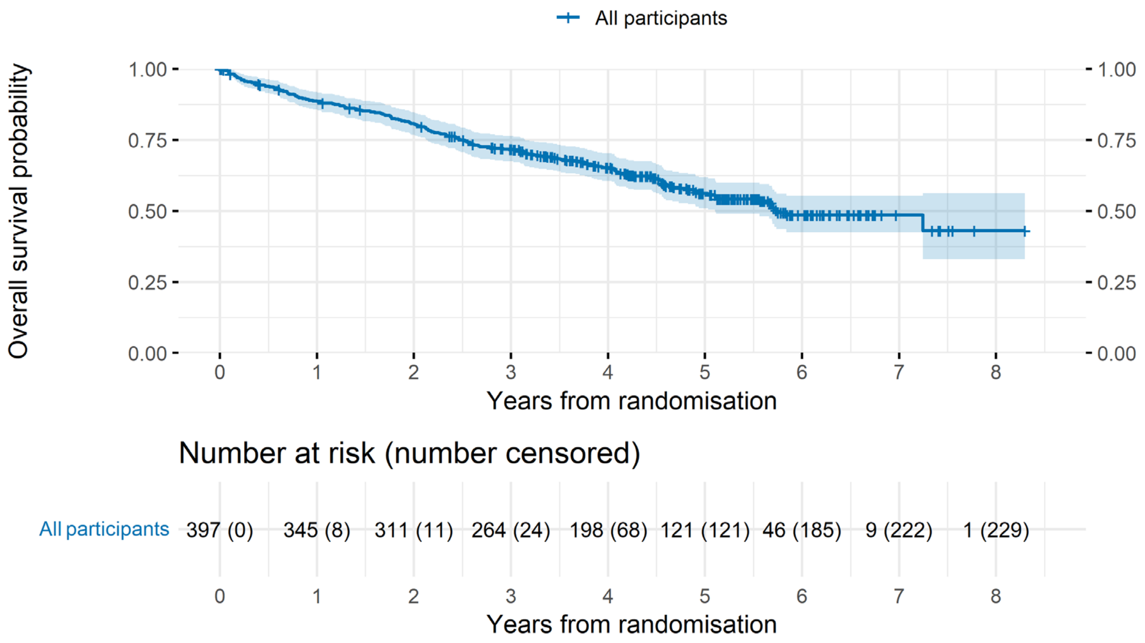

Figure S3: Assessment of the proportional hazards assumption for progression-free survival

Plots assessing the proportional hazards assumption. A) plot of log(-log(S(t)) for each treatment group. B and C) plots of Schoenfeld residuals. The assumption of proportional hazards appears to be supported by the Schoenfeld residuals being roughly zero over time and the log(-log(S(t)) curves appearing approximately parallel. We conclude that the proportional hazards assumption is not violated in such a way that would affect the interpretation of the primary analysis. CycleSchedule = Investigator choice of immunochemotherapy, 8x3 = R-CHOP and 6x4 = BR.

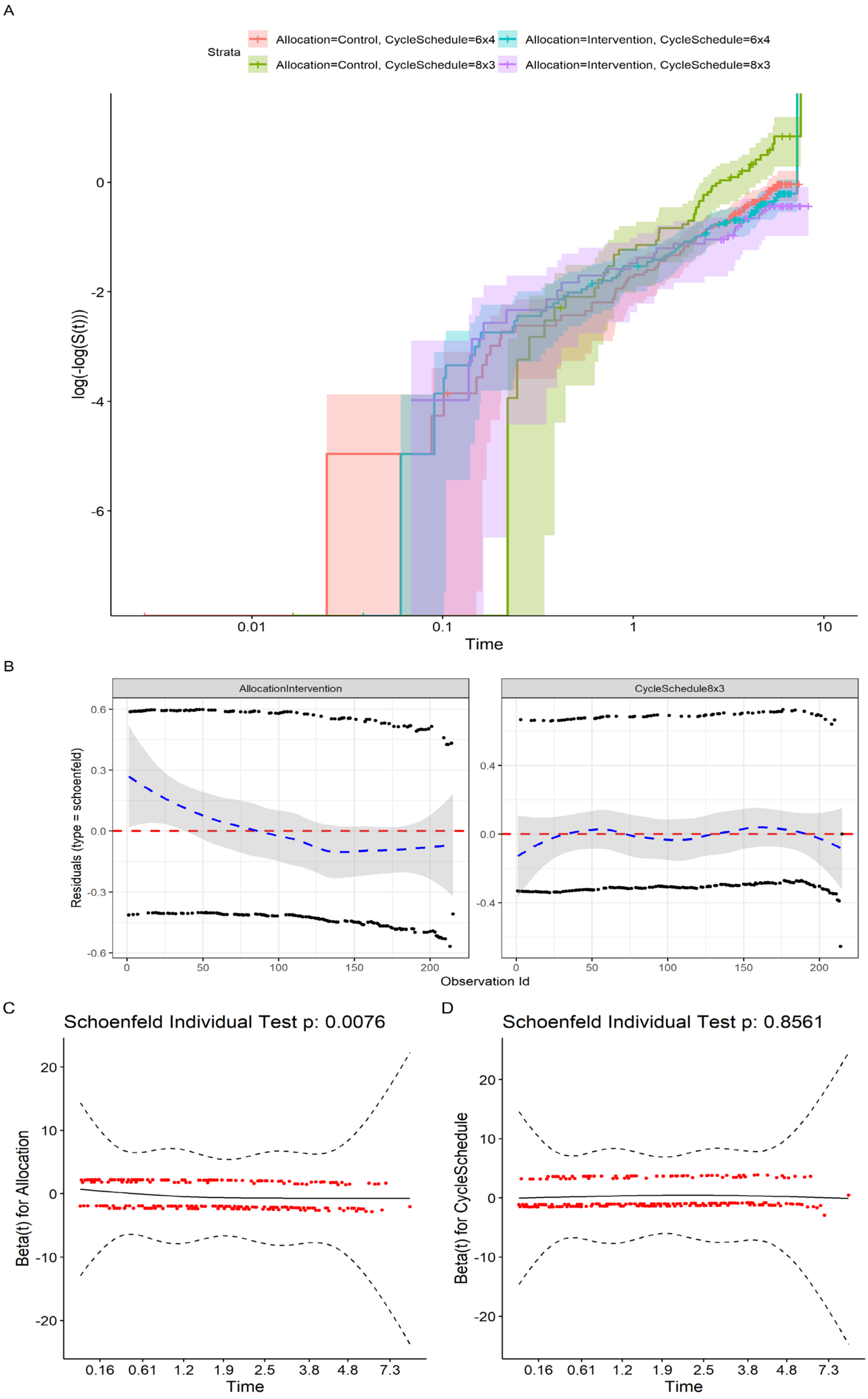
A
Strata
Allocation=Control, CycleSchedule=6x4
Allocation=Intervention, CycleSchedule=6x4
Allocation=Control, CycleSchedule=8x3
Allocation=Intervention, CycleSchedule=8x3
log(-log(S(t)))
Time
B
AllocationIntervention
CycleSchedule8x3
Residuals (type = schoenfeld)
Observation Id
C
Schoenfeld Individual Test p: 0.0076
Beta(t) for Allocation
Time
D
Schoenfeld Individual Test p: 0.8561
Beta(t) for CycleSchedule
Time

Figure S4: Progression-free survival by immunochemotherapy and treatment allocation

Kaplan-Meier plots of progression-free survival by pre-randomisation investigator choice of immunochemotherapy and treatment allocation, with 3- and 5-year progression-free survival rates with 95% confidence intervals.

3-year progression-free survival: Control – BR = 61.9% (95% CI 54.4% to 70.4%), Control – R-CHOP = 35.4% (95% CI 24.4% to 51.3%), Intervention – BR = 62.0% (95% CI 54.5% to 70.6%), Intervention – R-CHOP = 70.4% (95% CI 59.2% to 83.7%).

5-year progression-free survival: Control – BR = 47.4% (95% CI 39.5% to 56.9%), Control – R-CHOP = 19.2% (95% CI 10.6% to 35.1%), Intervention – BR = 50.8% (95% CI 42.8% to 60.4%), Intervention – R-CHOP = 52.4% (95% CI 40.0% to 68.6%).

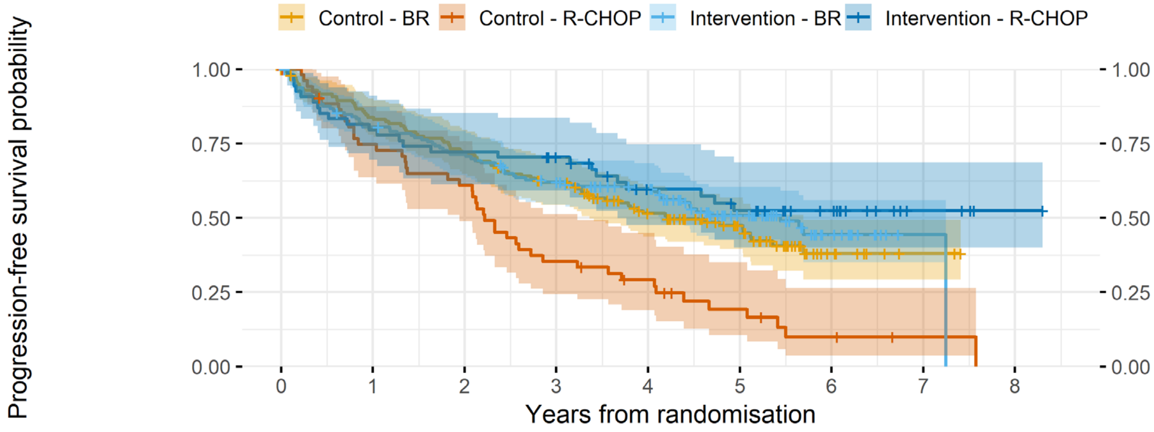

Number at risk (number censored)

	0	1	2	3	4	5	6	7	8
Control - BR	145 (0)	119 (3)	102 (3)	85 (6)	57 (21)	37 (37)	9 (59)	2 (66)	0 (68)
Control - R-CHOP	53 (0)	38 (2)	31 (2)	18 (2)	13 (4)	7 (6)	3 (7)	1 (9)	0 (9)
Intervention- BR	145 (0)	115 (2)	101 (3)	85 (6)	69 (19)	37 (42)	13 (63)	1 (75)	0 (75)
Intervention- R-CHOP	54 (0)	43 (0)	39 (0)	35 (3)	25 (8)	21 (9)	14 (16)	4 (26)	1 (29)

Years from randomisation

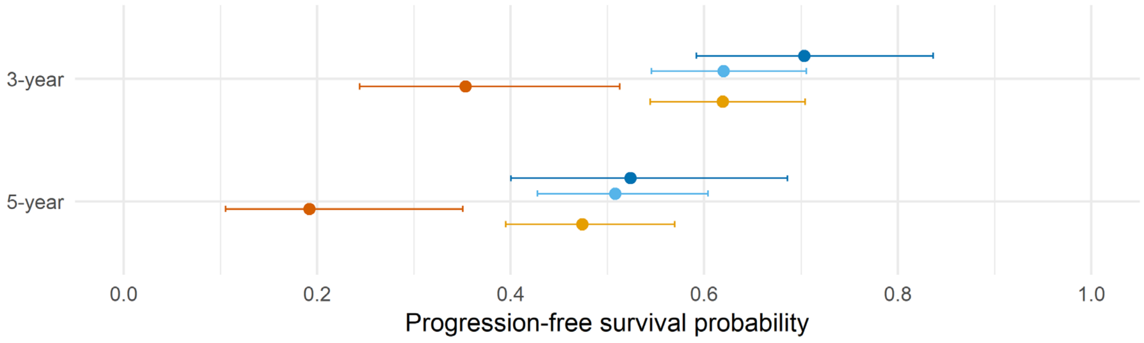

Figure S5: Progression-free survival by investigator choice of immunochemotherapy

Subgroup analysis of progression-free survival for subgroups with pre-randomisation investigator choice of immunochemotherapy R-CHOP and Bendamustine-rituximab. (P value for interaction between treatment effect and choice of chemotherapy = 0.0038)

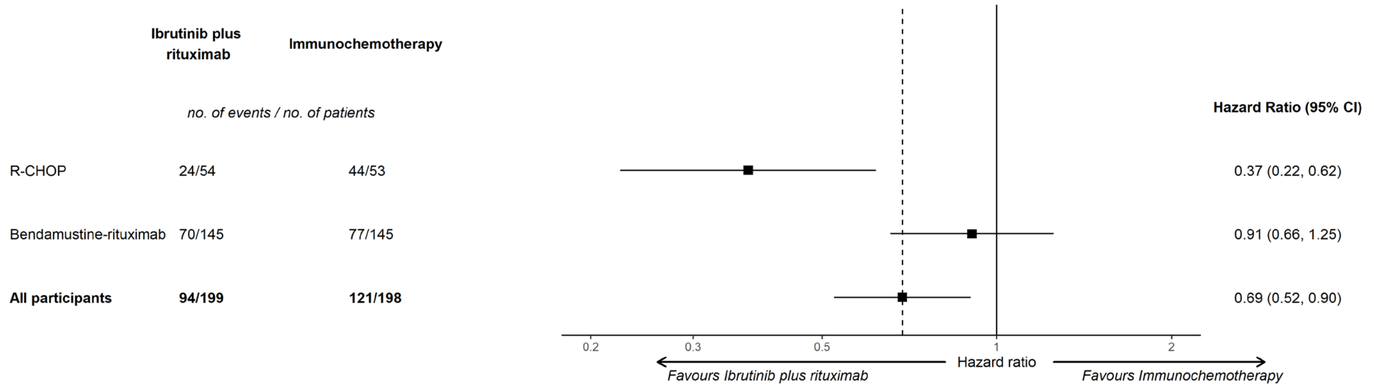

Figure S6: Kaplan-Meier plots of progression-free survival by TP53 mutation status

Kaplan-Meier plots of progression-free survival by TP53 mutation status and pre-randomisation investigator choice of immunochemotherapy (Fig A – all participants, Fig B – R-CHOP, Fig C – bendamustine-rituximab).

A: All participants

Mutated

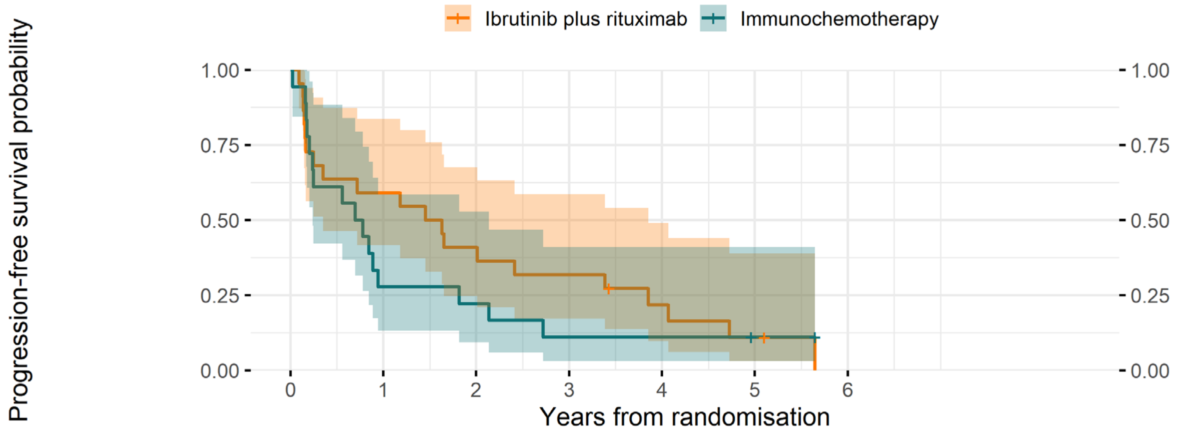

Number at risk (number censored)

	0	1	2	3	4	5	6
Ibrutinib plus rituximab	22 (0)	13 (0)	9 (0)	7 (0)	4 (1)	2 (1)	0 (2)
Immunochemotherapy	18 (0)	5 (0)	4 (0)	2 (0)	2 (0)	1 (1)	0 (2)

Years from randomisation

Unmutated

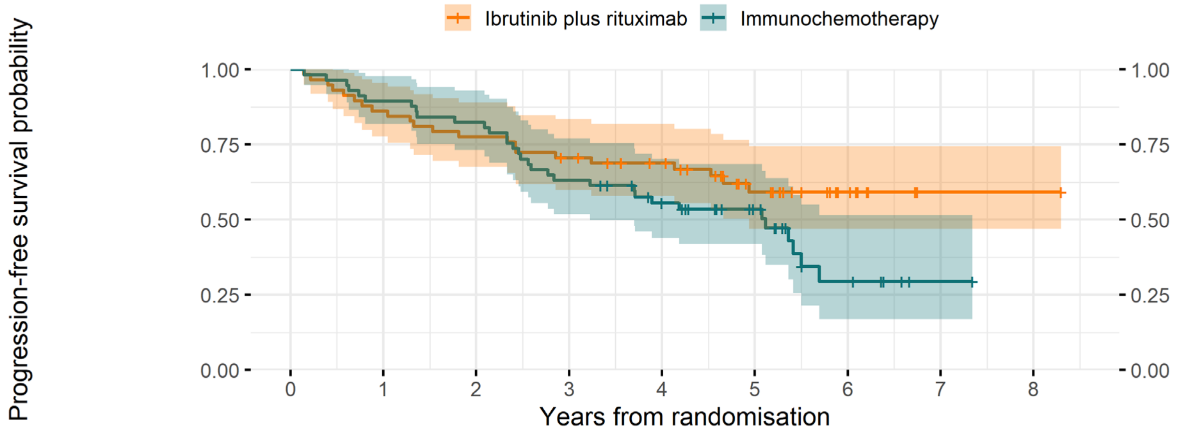

Number at risk (number censored)

	0	1	2	3	4	5	6	7	8
Ibrutinib plus rituximab	58 (0)	50 (0)	45 (0)	40 (1)	34 (6)	20 (16)	8 (28)	1 (35)	1 (35)
Immunochemotherapy	57 (0)	51 (0)	47 (0)	36 (0)	27 (5)	18 (13)	6 (19)	1 (24)	0 (25)

Years from randomisation

B: Immunochemotherapy choice of R-CHOP

Mutated

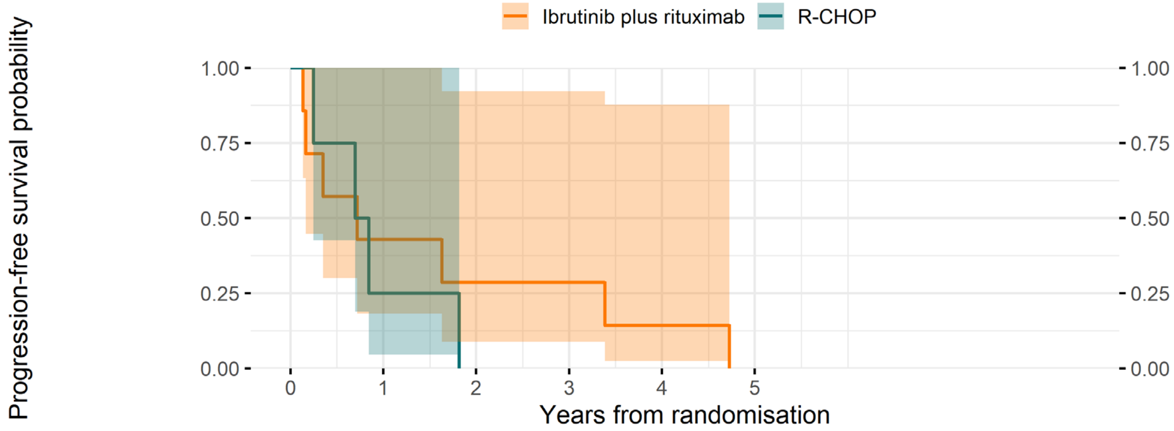

Number at risk (number censored)

	0	1	2	3	4	5
Ibrutinib plus rituximab	7 (0)	3 (0)	2 (0)	2 (0)	1 (0)	0 (0)
R-CHOP	4 (0)	1 (0)	0 (0)	0 (0)	0 (0)	0 (0)

Years from randomisation

Unmutated

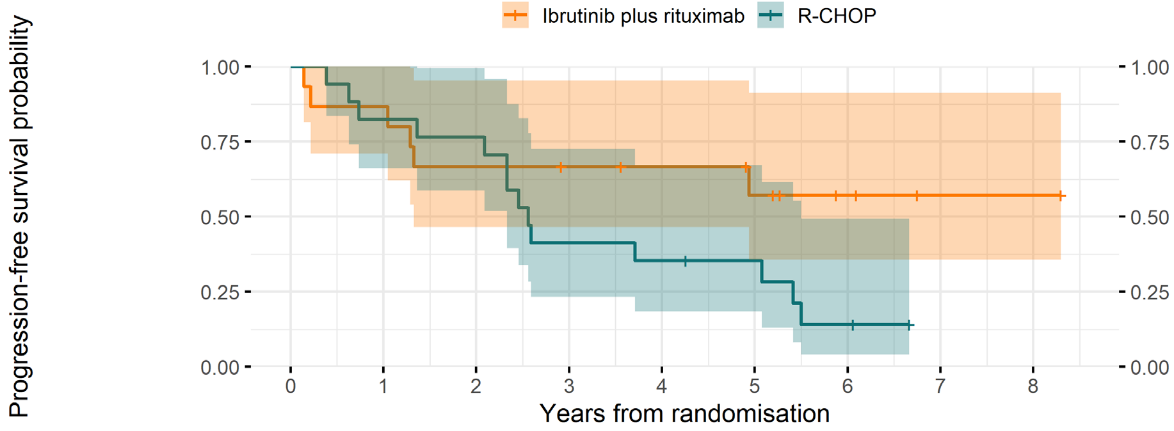

Number at risk (number censored)

	0	1	2	3	4	5	6	7	8
Ibrutinib plus rituximab	15 (0)	13 (0)	10 (0)	9 (1)	8 (2)	6 (3)	3 (6)	1 (8)	1 (8)
R-CHOP	17 (0)	14 (0)	13 (0)	7 (0)	6 (0)	5 (1)	2 (1)	0 (3)	0 (3)

Years from randomisation

C: Immunochemotherapy choice of Bendamustine-rituximab

Mutated

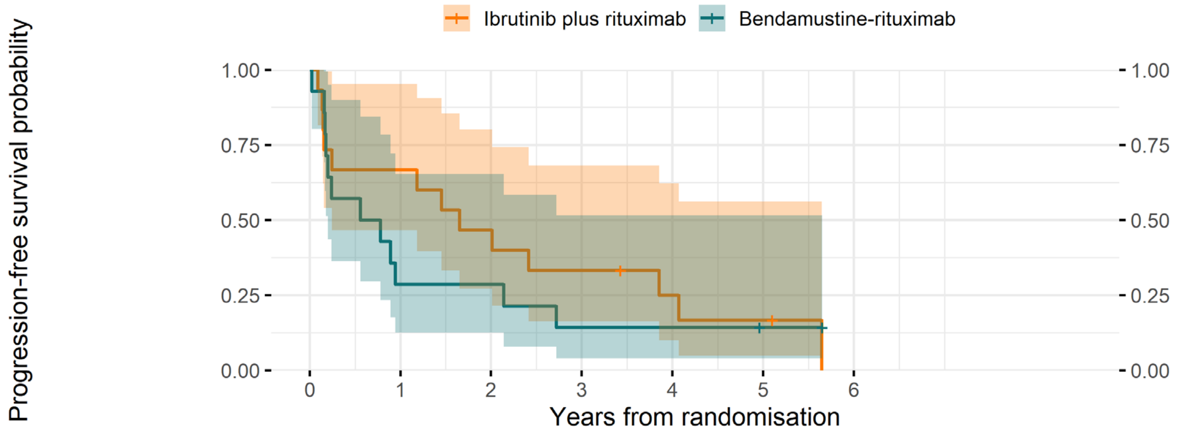

Number at risk (number censored)

	0	1	2	3	4	5	6
Ibrutinib plus rituximab	15 (0)	10 (0)	7 (0)	5 (0)	3 (1)	2 (1)	0 (2)
Bendamustine-rituximab	14 (0)	4 (0)	4 (0)	2 (0)	2 (0)	1 (1)	0 (2)

Years from randomisation

Unmutated

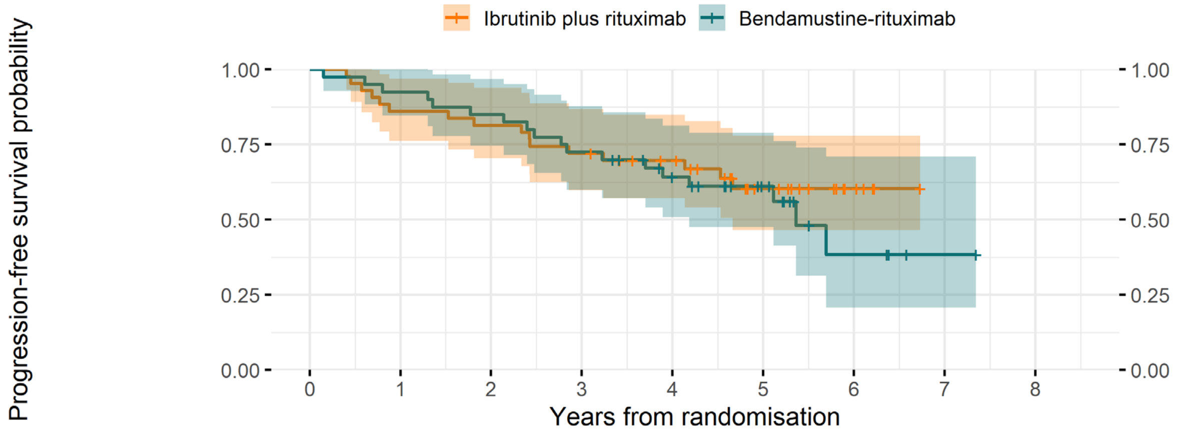

Number at risk (number censored)

	0	1	2	3	4	5	6	7	8
Ibrutinib plus rituximab	43 (0)	37 (0)	35 (0)	31 (0)	26 (4)	14 (13)	5 (22)	0 (27)	0 (27)
Bendamustine-rituximab	40 (0)	37 (0)	34 (0)	29 (0)	21 (5)	13 (12)	4 (18)	1 (21)	0 (22)

Years from randomisation

Figure S7: Kaplan-Meier plots of progression-free survival by blastoid status

Kaplan-Meier plots of progression-free survival by blastoid status and pre-randomisation investigator choice of immunochemotherapy (Fig A – all participants, Fig B – R-CHOP, Fig C – bendamustine-rituximab).

A

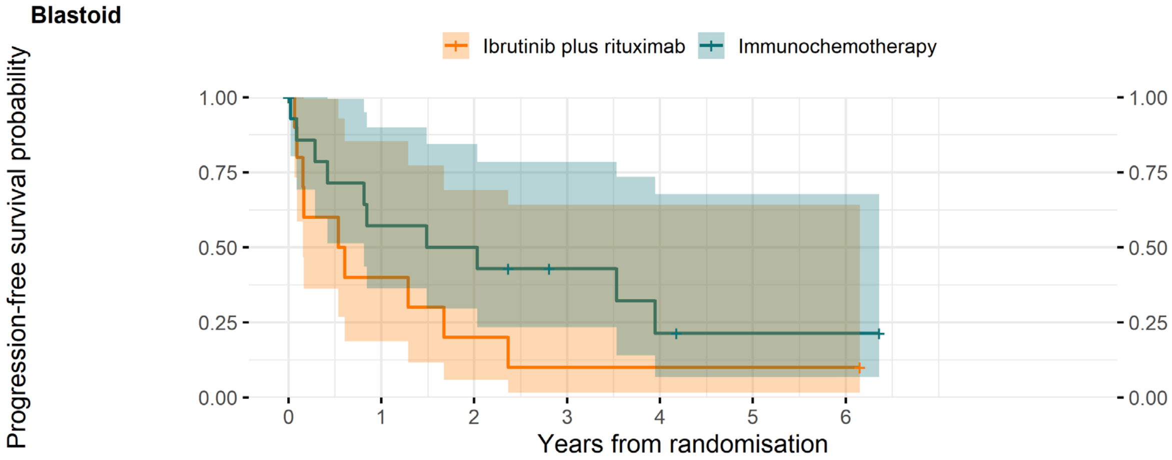

Number at risk (number censored)

	0	1	2	3	4	5	6
Ibrutinib plus rituximab	10 (0)	4 (0)	2 (0)	1 (0)	1 (0)	1 (0)	1 (0)
Immunochemotherapy	15 (0)	8 (1)	7 (1)	4 (3)	2 (3)	1 (4)	1 (4)

Years from randomisation

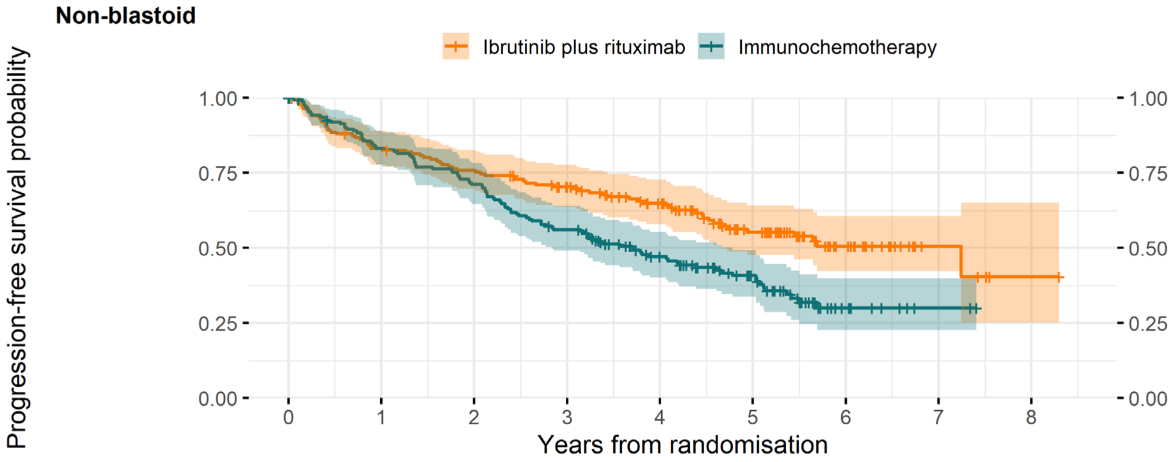

Number at risk (number censored)

	0	1	2	3	4	5	6	7	8
Ibrutinib plus rituximab	168 (0)	138 (2)	125 (3)	110 (9)	86 (25)	53 (47)	24 (73)	5 (92)	1 (95)
Immunochemotherapy	177 (0)	144 (4)	123 (4)	96 (5)	65 (22)	41 (38)	10 (60)	2 (68)	0 (70)

Years from randomisation

B

Blastoid

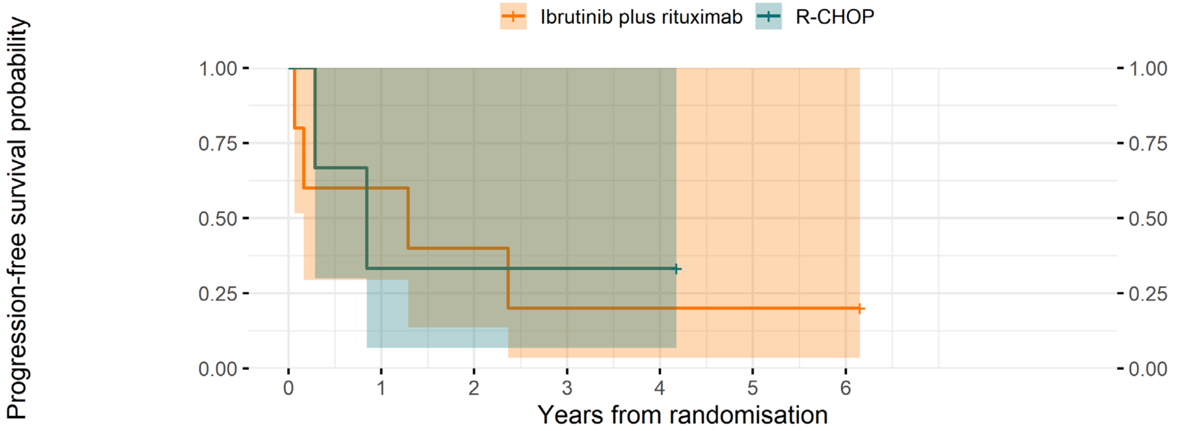

Number at risk (number censored)

	0	1	2	3	4	5	6
Ibrutinib plus rituximab	5 (0)	3 (0)	2 (0)	1 (0)	1 (0)	1 (0)	1 (0)
R-CHOP	3 (0)	1 (0)	1 (0)	1 (0)	1 (0)	0 (1)	0 (1)

Years from randomisation

Non-blastoid

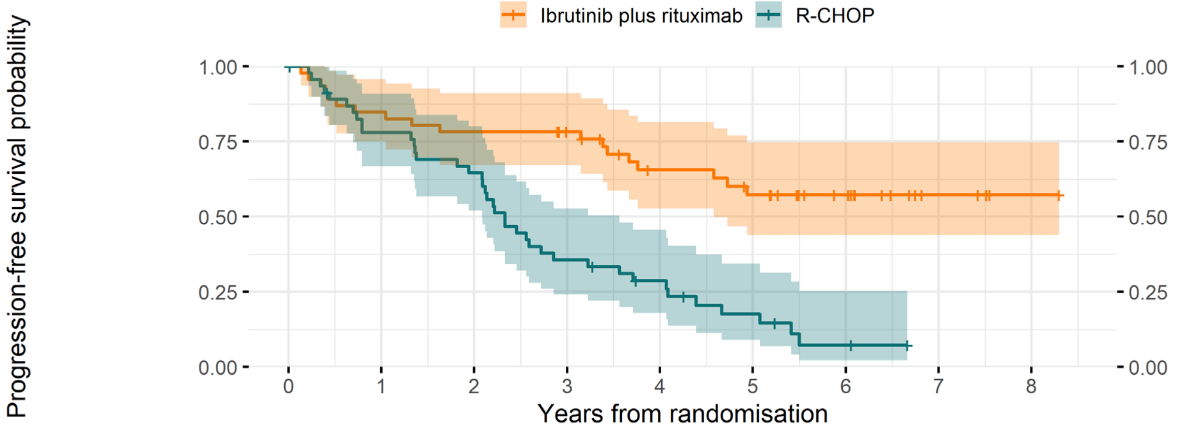

Number at risk (number censored)

	0	1	2	3	4	5	6	7	8
Ibrutinib plus rituximab	46 (0)	39 (0)	36 (0)	33 (3)	24 (7)	20 (8)	13 (15)	4 (24)	1 (27)
R-CHOP	47 (0)	35 (2)	29 (2)	16 (2)	11 (4)	6 (5)	2 (6)	0 (8)	0 (8)

Years from randomisation

C

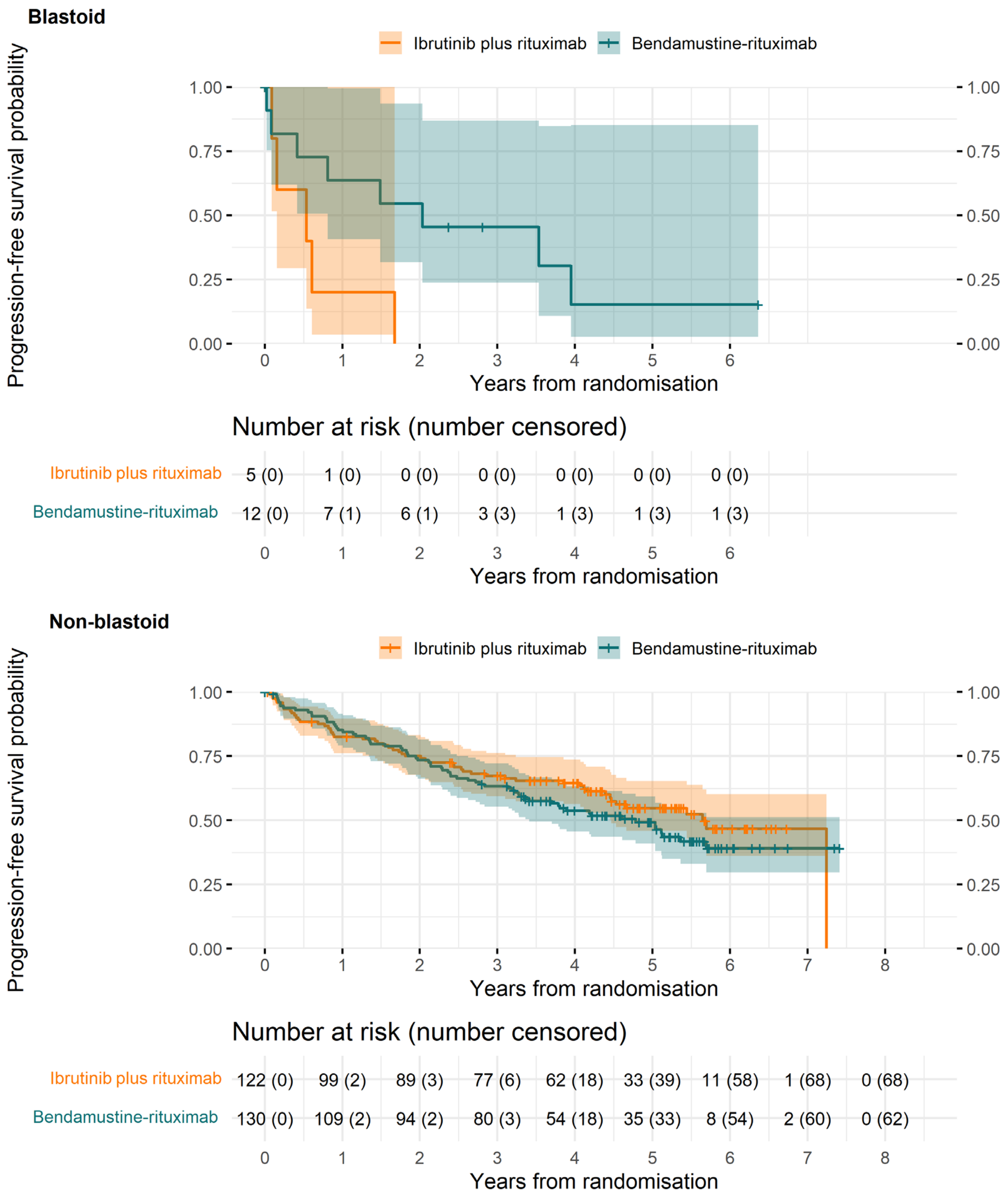
Blastoid
Ibrutinib plus rituximab
Bendamustine-rituximab
Progression-free survival probability
1.00
0.75
0.50
0.25
0.00
0 1 2 3 4 5 6
Years from randomisation
Number at risk (number censored)
Ibrutinib plus rituximab 5 (0) 1 (0) 0 (0) 0 (0) 0 (0) 0 (0) 0 (0)
Bendamustine-rituximab 12 (0) 7 (1) 6 (1) 3 (3) 1 (3) 1 (3) 1 (3)
0 1 2 3 4 5 6
Years from randomisation
Non-blastoid
Ibrutinib plus rituximab
Bendamustine-rituximab
Progression-free survival probability
1.00
0.75
0.50
0.25
0.00
0 1 2 3 4 5 6 7 8
Years from randomisation
Number at risk (number censored)
Ibrutinib plus rituximab 122 (0) 99 (2) 89 (3) 77 (6) 62 (18) 33 (39) 11 (58) 1 (68) 0 (68)
Bendamustine-rituximab 130 (0) 109 (2) 94 (2) 80 (3) 54 (18) 35 (33) 8 (54) 2 (60) 0 (62)
0 1 2 3 4 5 6 7 8
Years from randomisation

Figure S8: Kaplan-Meier plots of progression-free survival by Ki67 status

Kaplan-Meier plots of progression-free survival by Ki67 status (<30% and ≥30%) and pre-randomisation investigator choice of immunochemotherapy (Fig A – all participants, Fig B – R-CHOP, Fig C – bendamustine-rituximab).

A

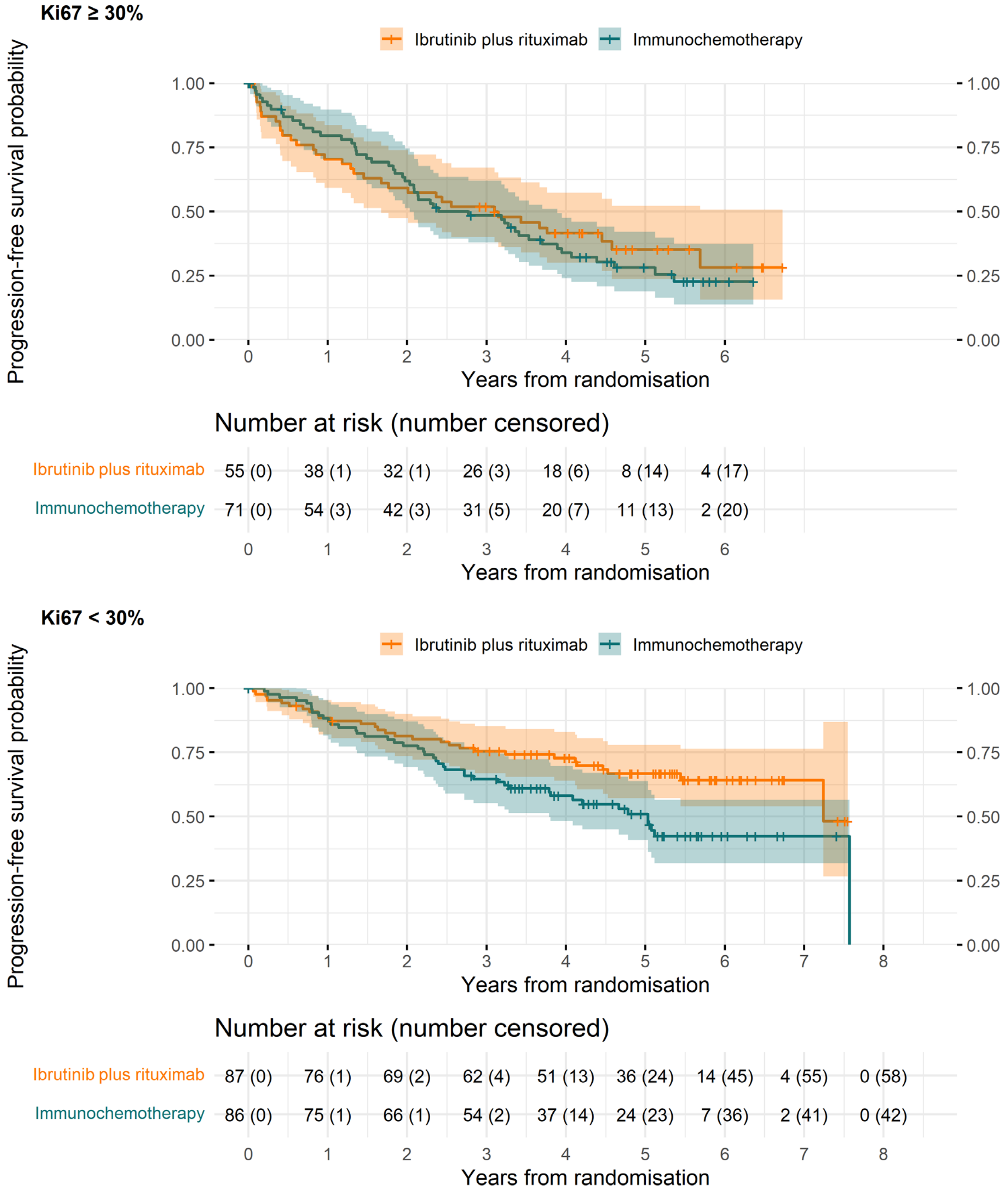

B

Ki67 ≥ 30%

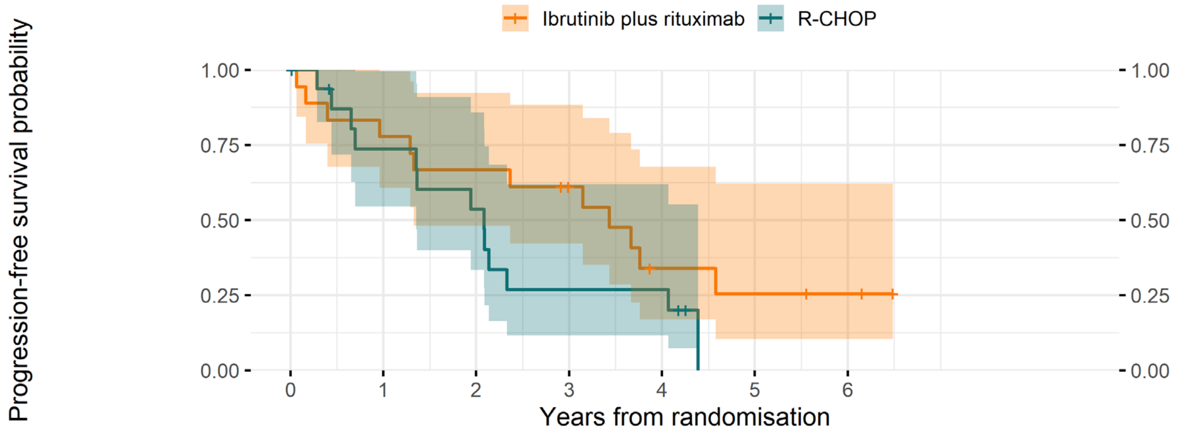

Number at risk (number censored)

	0	1	2	3	4	5	6
Ibrutinib plus rituximab	18 (0)	14 (0)	12 (0)	9 (2)	4 (3)	3 (3)	2 (4)
R-CHOP	17 (0)	11 (2)	8 (2)	4 (2)	4 (2)	0 (4)	0 (4)

Years from randomisation

Ki67 < 30%

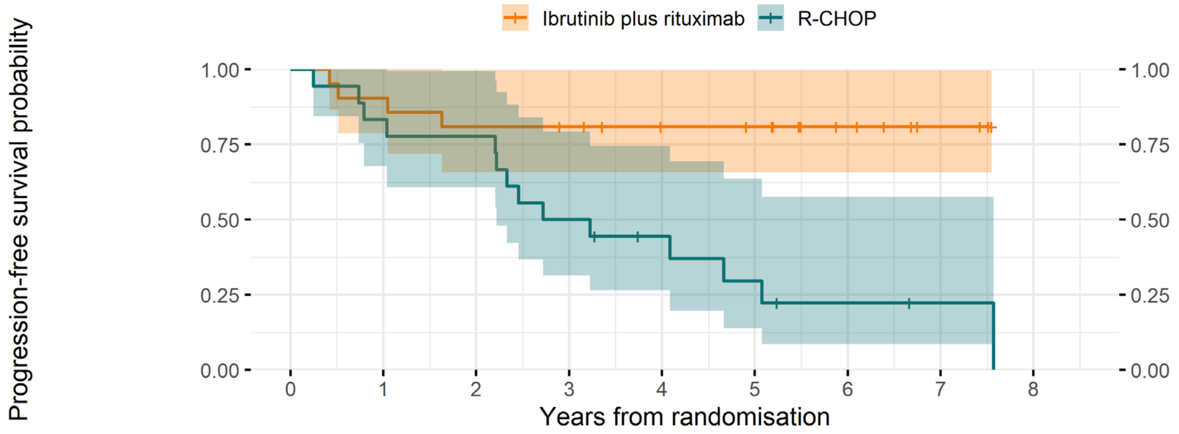

Number at risk (number censored)

	0	1	2	3	4	5	6	7	8
Ibrutinib plus rituximab	21 (0)	19 (0)	17 (0)	16 (1)	13 (4)	12 (5)	7 (10)	3 (14)	0 (17)
R-CHOP	18 (0)	15 (0)	14 (0)	9 (0)	6 (2)	4 (2)	2 (3)	1 (4)	0 (4)

Years from randomisation

C

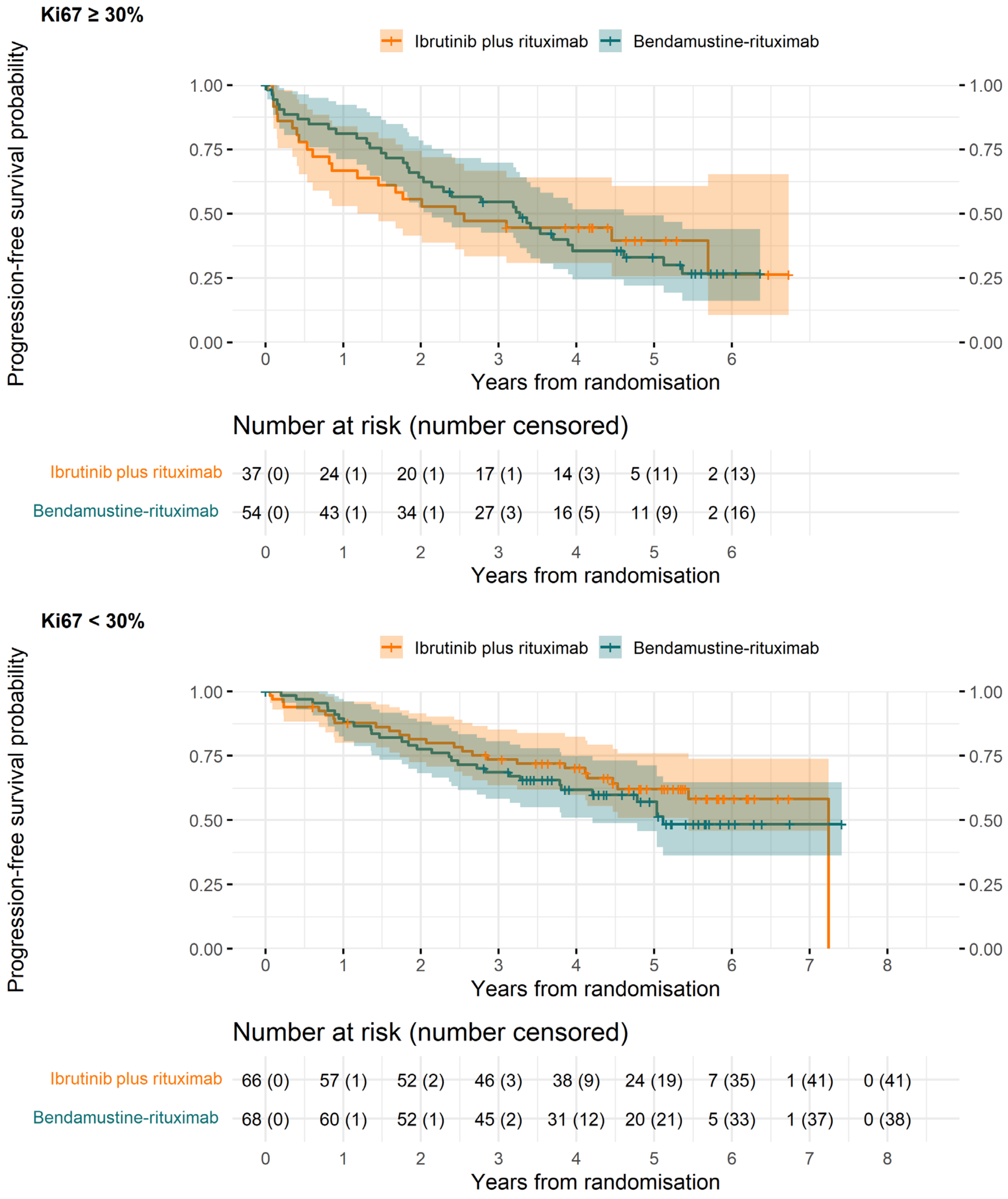
Ki67 ≥ 30%
Ibrutinib plus rituximab
Bendamustine-rituximab
Progression-free survival probability
1.00
0.75
0.50
0.25
0.00
0 1 2 3 4 5 6
Years from randomisation
Number at risk (number censored)
Ibrutinib plus rituximab 37 (0) 24 (1) 20 (1) 17 (1) 14 (3) 5 (11) 2 (13)
Bendamustine-rituximab 54 (0) 43 (1) 34 (1) 27 (3) 16 (5) 11 (9) 2 (16)
0 1 2 3 4 5 6
Years from randomisation
Ki67 < 30%
Ibrutinib plus rituximab
Bendamustine-rituximab
Progression-free survival probability
1.00
0.75
0.50
0.25
0.00
0 1 2 3 4 5 6 7 8
Years from randomisation
Number at risk (number censored)
Ibrutinib plus rituximab 66 (0) 57 (1) 52 (2) 46 (3) 38 (9) 24 (19) 7 (35) 1 (41) 0 (41)
Bendamustine-rituximab 68 (0) 60 (1) 52 (1) 45 (2) 31 (12) 20 (21) 5 (33) 1 (37) 0 (38)
0 1 2 3 4 5 6 7 8
Years from randomisation

Figure S9: Assessment of the proportional hazards assumption for overall survival

Plots assessing the proportional hazards assumption, including A) plot of log(log(-S(t)), B and C) plots of Schoenfeld residuals. We conclude that the proportionality of hazards assumption does not substantially change our interpretation. CycleSchedule = Investigator choice of immunochemotherapy, 8x3 = R-CHOP and 6x4 = BR.

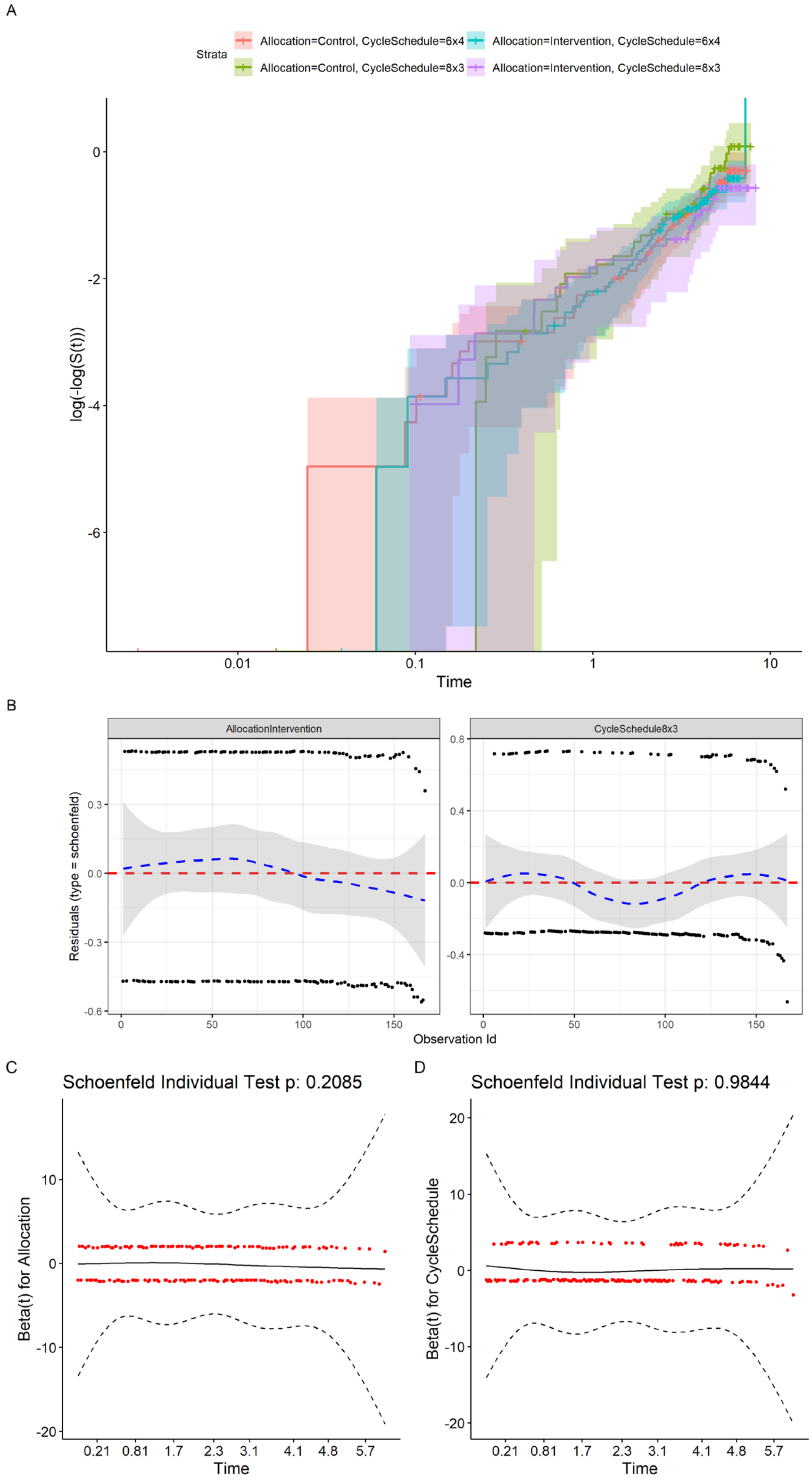

Figure S10: Causes of death by treatment allocation and pre-randomisation choice of immunochemotherapy

Pareto plot displaying the causes of death in a bar chart by most common to least common (left to right) with line graph representing cumulative deaths. (Fig A – all participants, Fig B – ibrutinib plus rituximab (choice of R-CHOP), Fig C – R-CHOP, Fig D – ibrutinib plus rituximab (choice bendamustine-rituximab), Fig E – bendamustine-rituximab).

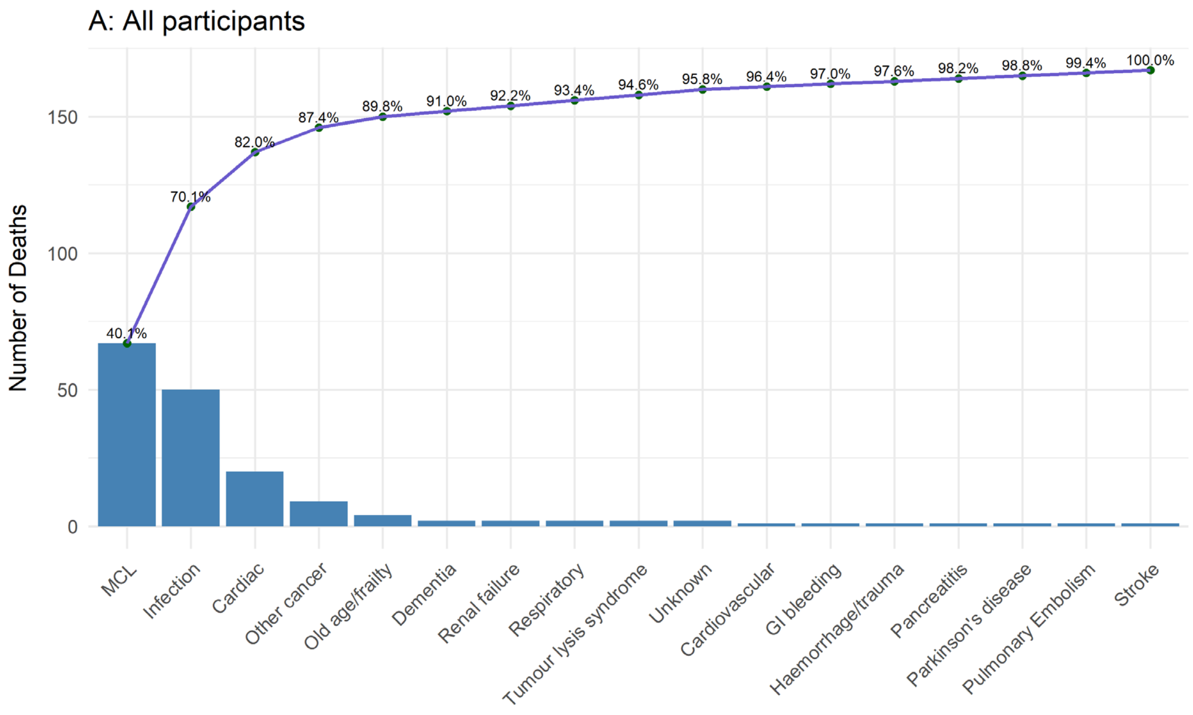

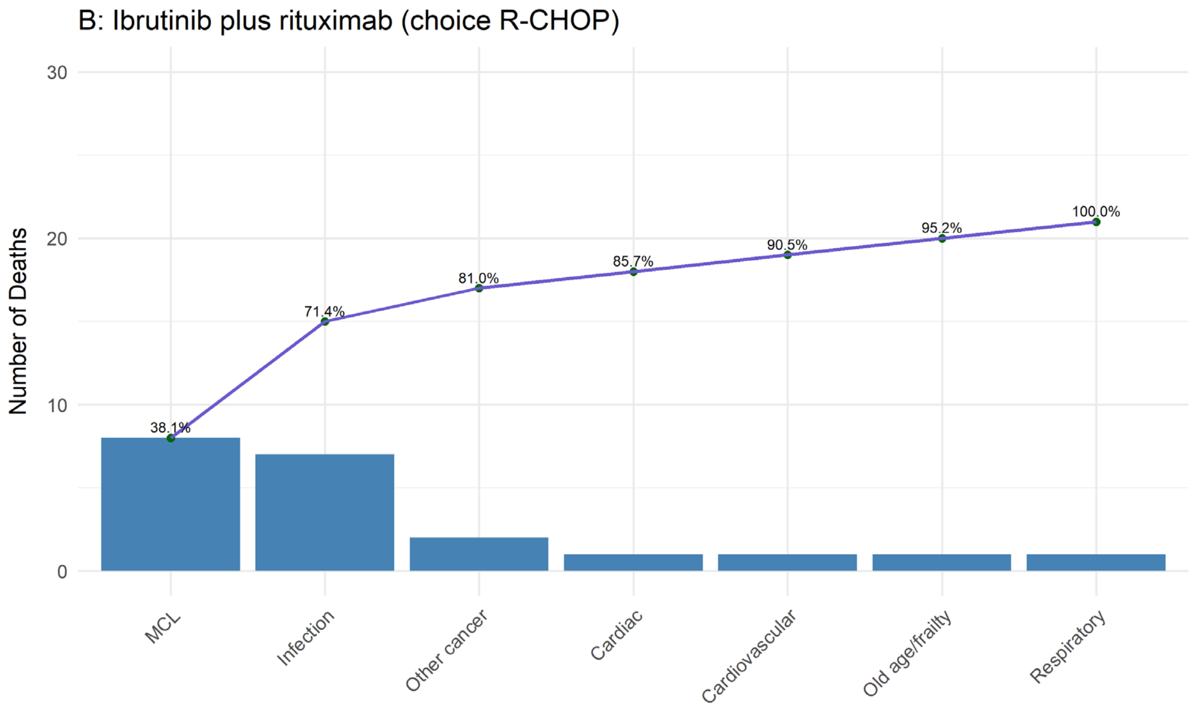

C: R-CHOP

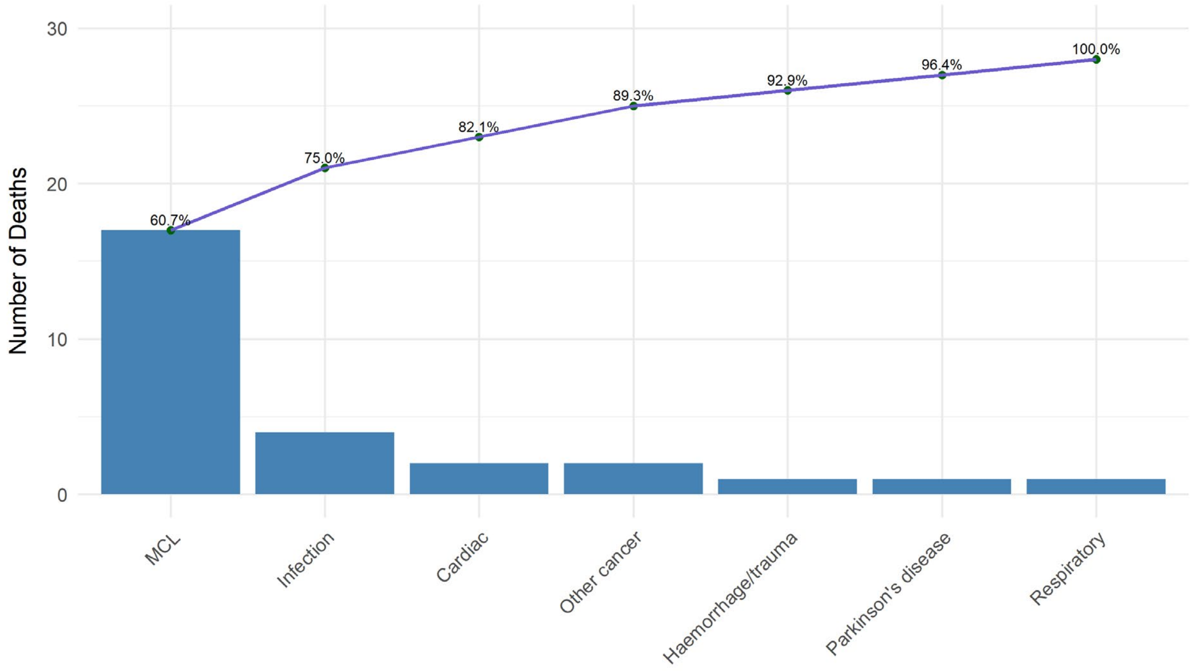

D: Ibrutinib plus rituximab (choice Bendamustine-rituximab)

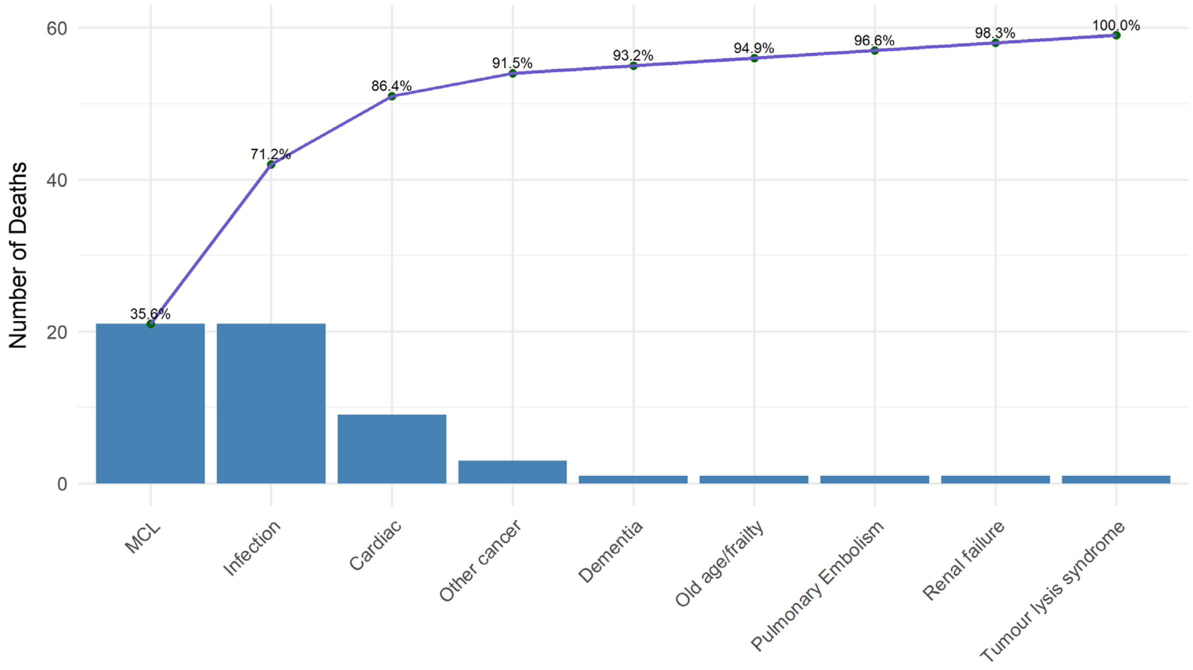

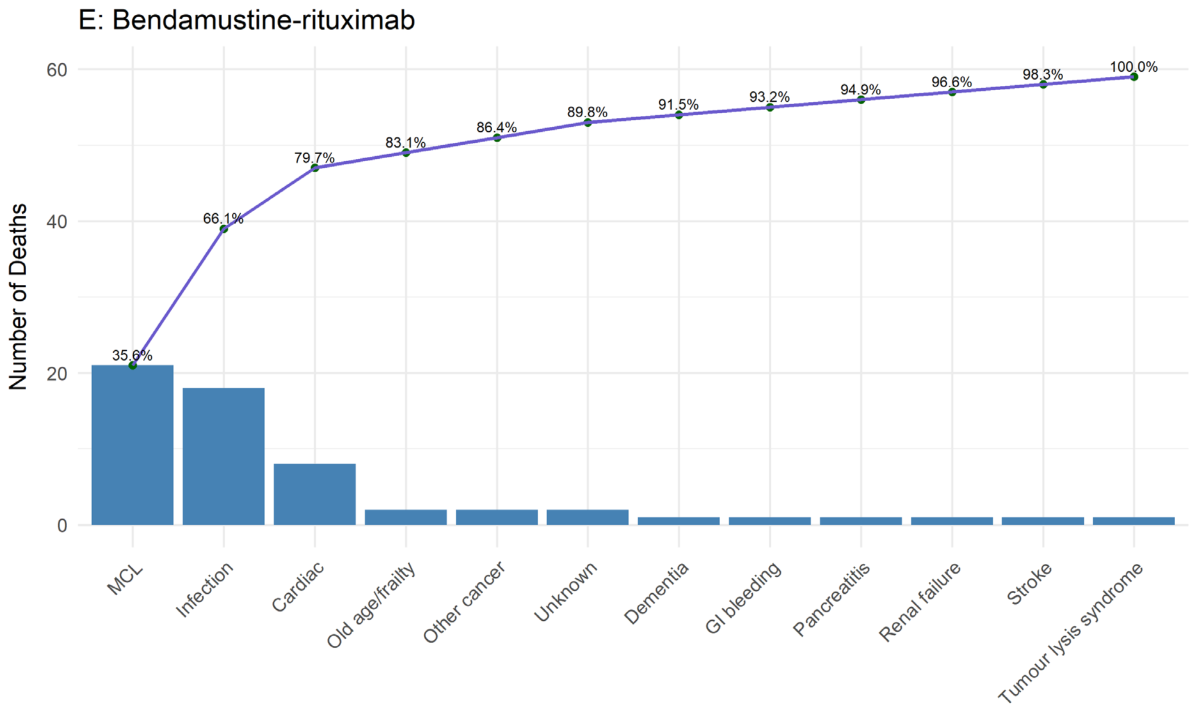
E: Bendamustine-rituximab
Number of Deaths
60
40
20
0
35.6%
66.1%
79.7%
83.1%
86.4%
89.8%
91.5%
93.2%
94.9%
96.6%
98.3%
100.0%
MCL
Infection
Cardiac
Old age/frailty
Other cancer
Unknown
Dementia
GI bleeding
Pancreatitis
Renal failure
Stroke
Tumour lysis syndrome

Figure S11: Quality of Life (EORTC QLQ-C30) scores by treatment taken (pre-randomisation investigator choice of immunochemotherapy and intervention)

European Organisation for Research and Treatment of Cancer quality of life questionnaire (EORTC QLQ-C30) scores range from 0 to 100, where a higher score represents a higher quality of life. A box plot is presented by time point, where the bold horizontal line marks the median, lower and upper edges of the box correspond to the first and third quartiles, the whiskers extend to the largest values within 1.5 times the IQR, and any points beyond this range are plotted as outliers. Summary statistics corresponding to this figure are given in Table S9.

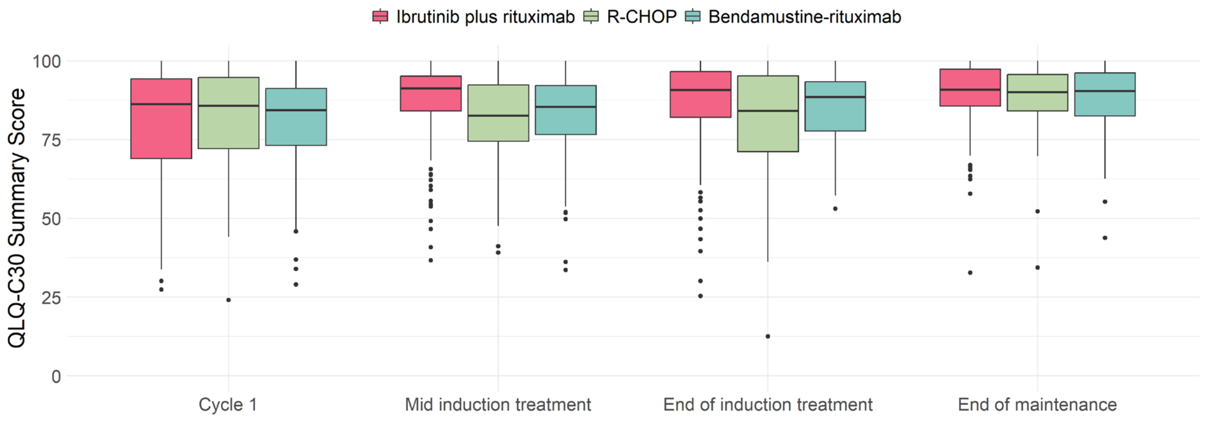

Figure S12: COVID-19 censored Kaplan-Meier plots for progression-free survival

Kaplan-Meier plots of progression-free survival with COVID-19 associated deaths censored at the final participant visit prior to COVID-19 death. Individual plots are presented by pre-randomisation investigator choice of immunochemotherapy (Fig A – all participants, Fig B – R-CHOP, Fig C – bendamustine-rituximab).

A

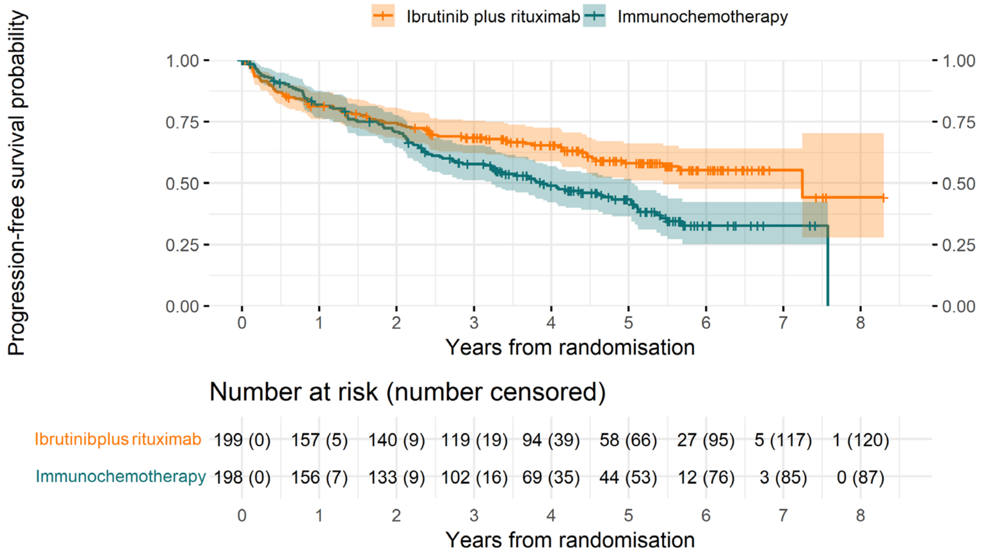
Ibrutinib plus rituximab
Immunochemotherapy
Progression-free survival probability
1.00
0.75
0.50
0.25
0.00
0 1 2 3 4 5 6 7 8
Years from randomisation
Number at risk (number censored)
Ibrutinibplus rituximab 199 (0) 157 (5) 140 (9) 119 (19) 94 (39) 58 (66) 27 (95) 5 (117) 1 (120)
Immunochemotherapy 198 (0) 156 (7) 133 (9) 102 (16) 69 (35) 44 (53) 12 (76) 3 (85) 0 (87)
0 1 2 3 4 5 6 7 8
Years from randomisation

B

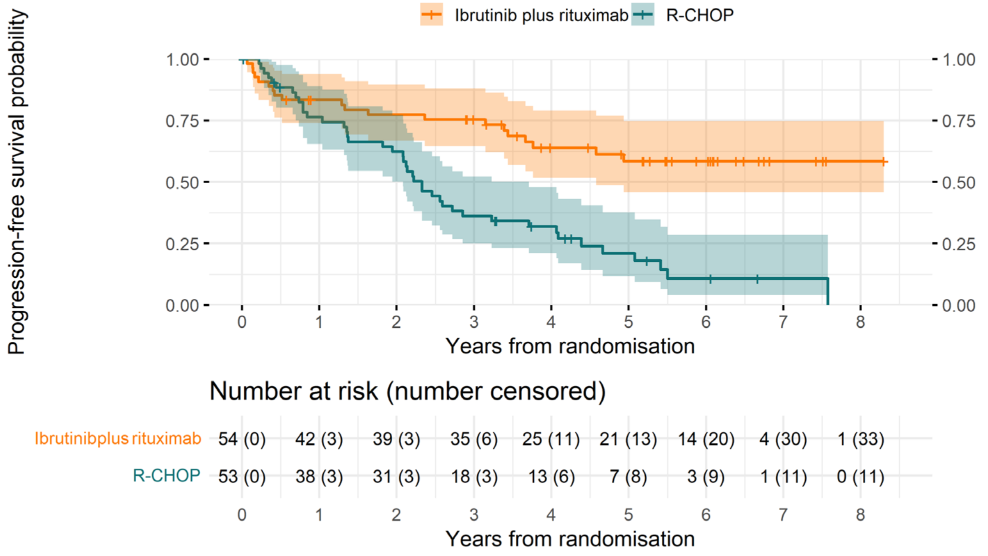
Ibrutinib plus rituximab
R-CHOP
Progression-free survival probability
1.00
0.75
0.50
0.25
0.00
0 1 2 3 4 5 6 7 8
Years from randomisation
Number at risk (number censored)
Ibrutinibplus rituximab 54 (0) 42 (3) 39 (3) 35 (6) 25 (11) 21 (13) 14 (20) 4 (30) 1 (33)
R-CHOP 53 (0) 38 (3) 31 (3) 18 (3) 13 (6) 7 (8) 3 (9) 1 (11) 0 (11)
0 1 2 3 4 5 6 7 8
Years from randomisation

C

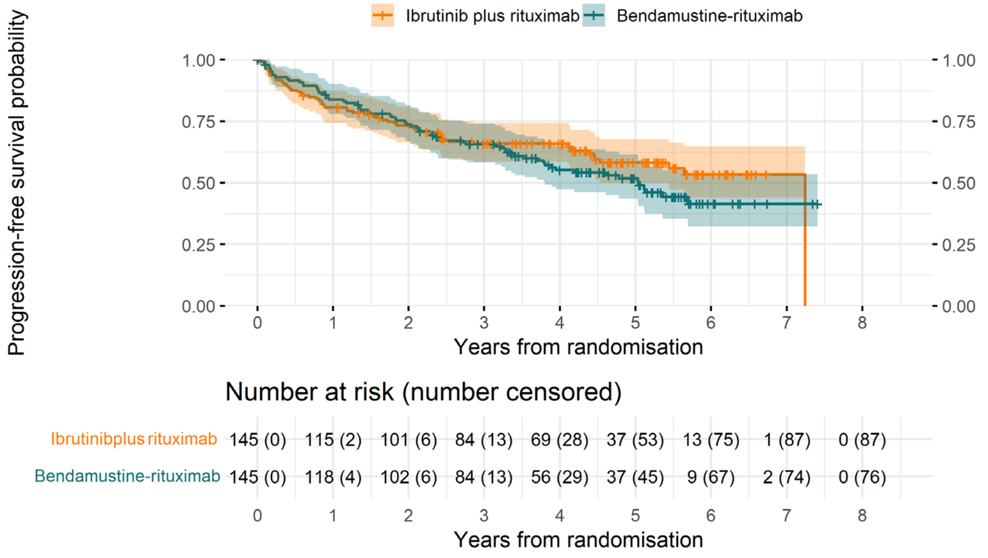
Ibrutinib plus rituximab
Bendamustine-rituximab
Progression-free survival probability
1.00
0.75
0.50
0.25
0.00
0 1 2 3 4 5 6 7 8
Years from randomisation
Number at risk (number censored)
Ibrutinibplus rituximab 145 (0) 115 (2) 101 (6) 84 (13) 69 (28) 37 (53) 13 (75) 1 (87) 0 (87)
Bendamustine-rituximab 145 (0) 118 (4) 102 (6) 84 (13) 56 (29) 37 (45) 9 (67) 2 (74) 0 (76)
0 1 2 3 4 5 6 7 8
Years from randomisation

Figure S13: COVID-19 censored Kaplan-Meier plots for overall survival

Kaplan-Meier plots of overall survival with COVID-19 associated deaths censored at the final participant visit prior to COVID-19 death. Individual plots are presented by pre-randomisation investigator choice of immunochemotherapy (Fig A – all participants, Fig B – R-CHOP, Fig C – Bendamustine-rituximab).

A

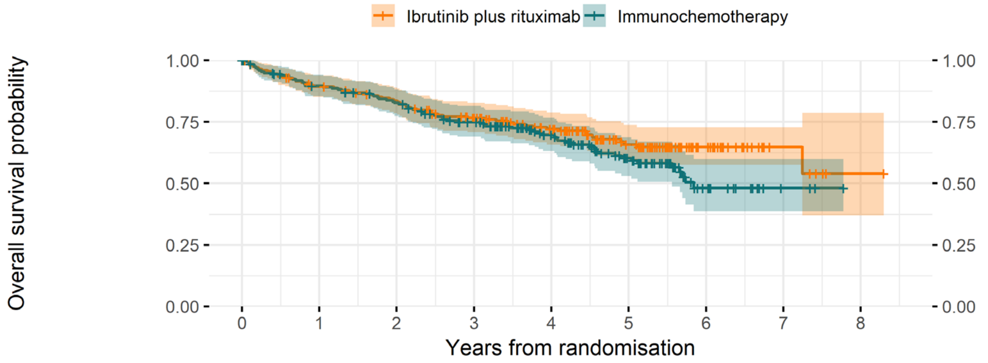

Number at risk (number censored)

	0	1	2	3	4	5	6	7	8
Ibrutinibplus rituximab	199 (0)	173 (5)	158 (9)	132 (22)	103 (44)	62 (78)	28 (111)	6 (133)	1 (137)
Immunochemotherapy	198 (0)	170 (8)	153 (12)	128 (23)	93 (50)	59 (73)	18 (107)	3 (122)	0 (125)

Years from randomisation

B

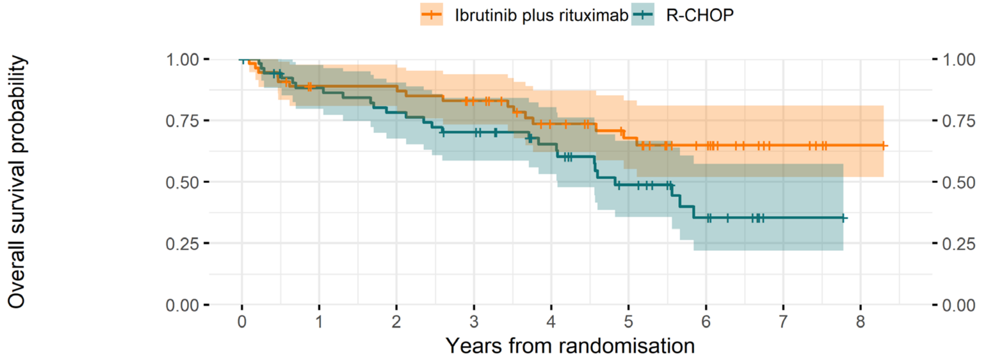

Number at risk (number censored)

	0	1	2	3	4	5	6	7	8
Ibrutinibplus rituximab	54 (0)	45 (3)	45 (3)	39 (6)	29 (12)	23 (16)	15 (23)	5 (33)	1 (37)
R-CHOP	53 (0)	44 (3)	39 (3)	34 (4)	26 (10)	16 (14)	8 (19)	1 (26)	0 (27)

Years from randomisation

C

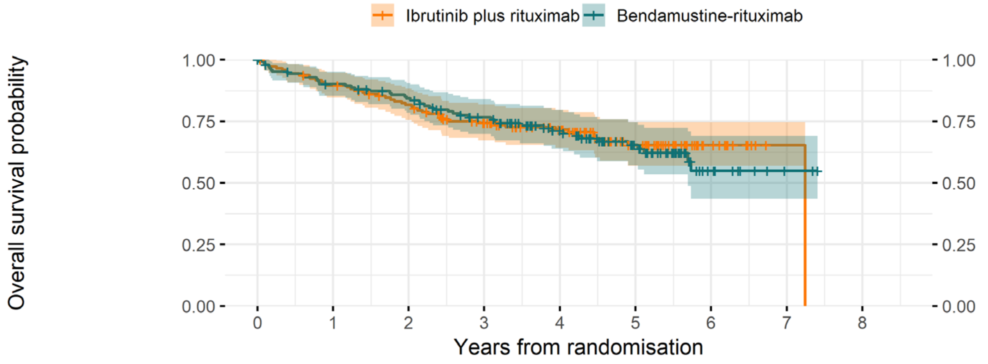

Number at risk (number censored)

	0	1	2	3	4	5	6	7	8
Ibrutinibplus rituximab	145 (0)	128 (2)	113 (6)	93 (16)	74 (32)	39 (62)	13 (88)	1 (100)	0 (100)
Bendamustine-rituximab	145 (0)	126 (5)	114 (9)	94 (19)	67 (40)	43 (59)	10 (88)	2 (96)	0 (98)

Years from randomisation

Table S1 Baseline participant characteristics by pre-randomization investigator choice of immunochemotherapy and treatment allocation of the 25 participants with blastoid disease.

	Bendamustine-rituximab		R-CHOP		Overall	
	**Bendamustine -rituximab,** N = 12	**Ibrutinib plus rituximab,** N = 5	**R-CHOP,** N = 3	**Ibrutinib plus rituximab,** N = 5	**Immunochemotherapy,** N = 15	**Ibrutinib plus rituximab,** N = 10
**Age (years)**						
Median (IQR)	75 (73, 80)	74 (72, 74)	76 (73, 78)	78 (75, 81)	75 (72, 79)	74 (72, 78)
[60, 70)	2 / 12 (17%)	1 / 5 (20%)	1 / 3 (33%)	1 / 5 (20%)	3 / 15 (20%)	2 / 10 (20%)
[70, 80)	7 / 12 (58%)	4 / 5 (80%)	2 / 3 (67%)	2 / 5 (40%)	9 / 15 (60%)	6 / 10 (60%)
[80, 90)	3 / 12 (25%)	0	0	2 / 5 (40%)	3 / 15 (20%)	2 / 10 (20%)
**Gender**						
Male	7 / 12 (58%)	1 / 5 (20%)	1 / 3 (33%)	4 / 5 (80%)	8 / 15 (53%)	5 / 10 (50%)
Female	5 / 12 (42%)	4 / 5 (80%)	2 / 3 (67%)	1 / 5 (20%)	7 / 15 (47%)	5 / 10 (50%)
**ECOG**						
0	6 / 12 (50%)	2 / 5 (40%)	1 / 3 (33%)	0	7 / 15 (47%)	2 / 10 (20%)
1	4 / 12 (33%)	0	2 / 3 (67%)	4 / 5 (80%)	6 / 15 (40%)	4 / 10 (40%)
2	2 / 12 (17%)	3 / 5 (60%)	0	1 / 5 (20%)	2 / 15 (13%)	4 / 10 (40%)
**Disease staging**						
II	2 / 12 (17%)	0	0	0	2 / 15 (13%)	0
III	0	0	0	1 / 5 (20%)	0	1 / 10 (10%)
IV	10 / 12 (83%)	5 / 5 (100%)	3 / 3 (100%)	4 / 5 (80%)	13 / 15 (87%)	9 / 10 (90%)

	Bendamustine-rituximab		R-CHOP		Overall	
	**Bendamustine -rituximab**, N = 12	**Ibrutinib plus rituximab**, N = 5	**R-CHOP**, N = 3	**Ibrutinib plus rituximab**, N = 5	**Immunochemotherapy**, N = 15	**Ibrutinib plus rituximab**, N = 10
**Ki67 ≥ 30**	11 / 12 (92%)	4 / 4 (100%)	2 / 2 (100%)	5 / 5 (100%)	13 / 14 (93%)	9 / 9 (100%)
Missing	0	1	1	0	1	1
**MIPI risk category**						
Low	0	0	0	0	0	0
Intermediate	5 / 11 (45%)	1 / 5 (20%)	1 / 3 (33%)	1 / 5 (20%)	6 / 14 (43%)	2 / 10 (20%)
High	6 / 11 (55%)	4 / 5 (80%)	2 / 3 (67%)	4 / 5 (80%)	8 / 14 (57%)	8 / 10 (80%)
Missing	1	0			1	0
**TP53 mutation**	1 / 2 (50%)	2 / 2 (100%)	1 / 1 (100%)	1 / 2 (50%)	2 / 3 (67%)	3 / 4 (75%)
Missing	10	3	2	3	12	6
**Country**						
Nordic	4 / 12 (33%)	1 / 5 (20%)	0	1 / 5 (20%)	4 / 15 (27%)	2 / 10 (10%)
UK	8 / 12 (67%)	4 / 5 (80%)	3 / 3 (10%)	4 / 5 (80%)	11 / 15 (73%)	8 / 10 (80%)

Table S2: Supplementary and sensitivity analyses

Cox proportional hazards model results for intervention/control (hazard ratio, HR, and 95% confidence intervals, CI) for progression-free survival (PFS), overall survival (OS), failure-free survival (FFS) and time-to-next treatment. Adjusted analysis includes adjustment for pre-randomisation investigator choice of chemotherapy. The pre-protocol analysis consists of participants who do not discontinue treatment prior to mid-induction treatment and have a mid-treatment scan but excludes participants in the control group with a mid-induction treatment scan of stable disease who do not discontinue treatment. Failure-free survival represents a modified progression-free survival, incorporating stable disease at mid-treatment and the start of a further MCL treatment as events. Events contributing to next treatment are second line MCL treatment or death. The COVID-19 analysis censors participants with COVID-19 related deaths at their final visit prior to death.

Outcome		Ibrutinib plus rituximab / Immunochemotherapy; HR (95% CI)	Ibrutinib plus rituximab / R-CHOP; HR (95% CI)	Ibrutinib plus rituximab / Bendamustine-rituximab; HR (95% CI)
PFS	Adjusted	0.69 (0.52, 0.90)	-	-
	Unadjusted	0.70 (0.54, 0.92)	0.37 (0.22, 0.62)	0.91 (0.66, 1.25)
	Per-protocol (adjusted)	0.71 (0.53, 0.93)	-	-
	Per-protocol (unadjusted)	0.73 (0.55, 0.96)	0.38 (0.23, 0.63)	0.96 (0.68, 1.34)
	COVID-19 (adjusted)	0.62 (0.47, 0.83)	-	-
	COVID-19 (unadjusted)	0.64 (0.48, 0.85)	0.32 (0.19, 0.56)	0.84 (0.59, 1.19)
OS	Adjusted	0.87 (0.64, 1.18)	-	-
	Unadjusted	0.87 (0.64, 1.18)	0.64 (0.36, 1.13)	1.00 (0.70, 1.44)
	Per-protocol (adjusted)	0.90 (0.66, 1.23)	-	-
	Per-protocol (unadjusted)	0.90 (0.66, 1.23)	0.64 (0.36, 1.13)	1.06 (0.73, 1.53)
	COVID-19 (adjusted)	0.79 (0.56, 1.11)	-	-
	COVID-19 (unadjusted)	0.79 (0.56, 1.12)	0.53 (0.28, 0.99)	0.96 (0.64, 1.44)
FFS	Adjusted	0.67 (0.52, 0.88)	-	-
	Unadjusted	0.69 (0.53, 0.90)	0.41 (0.25, 0.67)	0.85 (0.62, 1.18)
Next treatment	Adjusted	0.69 (0.52, 0.91)	-	-
	Unadjusted	0.70 (0.53, 0.92)	0.45 (0.27, 0.75)	0.85 (0.61, 1.18)

Table S3: Progressive disease timing

Timing of occurrence of progressive disease. If a participant progressed after discontinuing treatment they are off the study pathway. The denominator is the number of participants in each group that have documented disease progression.

Timing of progression	Bendamustine-rituximab		R-CHOP		Overall	
	Bendamustine-rituximab	Ibrutinib plus rituximab	R-CHOP	Ibrutinib plus rituximab	Immunochemotherapy	Ibrutinib plus rituximab
**Induction treatment**	6 / 44 (13.6%)	12 / 35 (34.3%)	3 / 33 (9.1%)	8 / 14 (57.1%)	9 / 77 (11.7%)	20 / 49 (40.8%)
**Maintenance**	18 / 44 (40.9%)	13 / 35 (37.1%)	18 / 33 (54.5%)	4 / 14 (28.6%)	36 / 77 (46.8%)	17 / 49 (34.7%)
**Follow-up**	11 / 44 (25.0%)	3 / 35 (8.6%)	7 / 33 (21.2%)	2 / 14 (14.3%)	18 / 77 (23.4%)	5 / 49 (10.2%)
**Off study pathway**	9 / 44 (20.5%)	7 / 35 (20.0%)	5 / 33 (15.2%)	0 / 14 (0.0%)	14 / 77 (18.2%)	7 / 49 (14.3%)

Table S4: Disease response by pre-randomisation choice of immunochemotherapy and treatment allocation.

Disease response was evaluated at mid-induction treatment, end of induction treatment, maintenance visit 3, maintenance visit 6, maintenance visit 9 and end of maintenance. Percentages are of the whole group, regardless of scan availability for assessment, i.e. including participants who withdrew, died or progressed prior to mid-induction treatment.

Of the four participants receiving BR with stable disease at mid-induction, two stopped treatment at this timepoint, and of the three receiving R-CHOP, two stopped treatment at this timepoint.

		Bendamustine-rituximab		R-CHOP		Overall	
		**Bendamustine-rituximab**	**Ibrutinib plus rituximab**	**R-CHOP**	**Ibrutinib plus rituximab**	**Immunochemotherapy**	**Ibrutinib plus rituximab**
**Best response**	**Complete response**	80/145 (55.2%)	86/145 (59.3%)	25/53 (47.2%)	21/54 (38.9%)	105/198 (53%)	107/199 (53.8%)
	**Complete response (unconfirmed)**	12/145 (8.3%)	9/145 (6.2%)	4/53 (7.5%)	6/54 (11.1%)	16/198 (8.1%)	15/199 (7.5%)
	**Partial response**	30/145 (20.7%)	30/145 (20.7%)	18/53 (34%)	19/54 (35.2%)	48/198 (24.2%)	49/199 (24.6%)
	**Stable disease**	4/145 (2.8%)	3/145 (2.1%)	2/53 (3.8%)	3/54 (5.6%)	6/198 (3%)	6/199 (3%)
	**Progressive disease**	3/145 (2.1%)	9/145 (6.2%)	1/53 (1.9%)	4/54 (7.4%)	4/198 (2%)	13/199 (6.5%)
	**Missing**	16	8	3	1	19	9
**Mid induction treatment response**	**Complete response**	36 / 145 (24.8%)	33 / 145 (22.8%)	9 / 53 (17.0%)	4 / 54 (7.4%)	45 / 198 (22.7%)	37 / 199 (18.6%)
	**Complete response (unconfirmed)**	5 / 145 (3.4%)	6 / 145 (4.1%)	1 / 53 (1.9%)	1 / 54 (1.9%)	6 / 198 (3.0%)	7 / 199 (3.5%)
	**Partial response**	76 / 145 (52.4%)	81 / 145 (55.9%)	36 / 53 (67.9%)	39 / 54 (72.2%)	112 / 198 (56.6%)	120 / 199 (60.3%)
	**Stable disease**	4 / 145 (2.8%)	4 / 145 (2.8%)	3 / 53 (5.7%)	4 / 54 (7.4%)	7 / 198 (3.5%)	8 / 199 (4.0%)
	**Progressive disease**	2 / 145 (1.4%)	1 / 145 (0.7%)	0 / 53 (0.0%)	0 / 54 (0.0%)	2 / 198 (1.0%)	1 / 199 (0.5%)
	**Missing**	22	20	4	6	26	26

Table S5: Causes of non-relapse mortality

Non-relapse mortality details the causes of death of participants without a prior progression event. Non-relapse deaths contribute to the progression-free survival endpoint.

Cause of death	Bendamustine-rituximab		R-CHOP		Overall	
	Bendamustine-rituximab	Ibrutinib plus rituximab	R-CHOP	Ibrutinib plus rituximab	Immunochemotherapy	Ibrutinib plus rituximab
**MCL**	2 / 33 (6.1%)	2 / 35 (5.7%)	3 / 11 (27.3%)	0	5 / 44 (11.4%)	2 / 45 (4.4%)
**Infection**	14 / 33 (42.4%)	18 / 35 (51.4%)	4 / 11 (36.4%)	5 / 10 (50.0%)	18 / 44 (40.9%)	23 / 45 (51.1%)
**Cardiac**	6 / 33 (18.2%)	9 / 35 (25.7%)	2 / 11 (18.2%)	0	8 / 44 (18.2%)	9 / 45 (20.0%)
**Other cancer**	2 / 33 (6.1%)	2 / 35 (5.7%)	1 / 11 (9.1%)	2 / 10 (20.0%)	3 / 44 (6.8%)	4 / 45 (8.9%)
**Old age/frailty**	2 / 33 (6.1%)	1 / 35 (2.9%)	0	1 / 10 (10.0%)	2 / 44 (4.5%)	2 / 45 (4.4%)
**Dementia**	1 / 33 (3.0%)	1 / 35 (2.9%)	0	0	1 / 44 (2.3%)	1 / 45 (2.2%)
**Renal failure**	1 / 33 (3.0%)	1 / 35 (2.9%)	0	0	1 / 44 (2.3%)	1 / 45 (2.2%)
**Respiratory**	0	0	0	1 / 10 (10.0%)	0	1 / 45 (2.2%)
**Tumour lysis syndrome**	1 / 33 (3.0%)	0	0	0	1 / 44 (2.3%)	0
**Unknown**	1 / 33 (3.0%)	0	0	0	1 / 44 (2.3%)	0
**Cardiovascular**	0	0	0	1 / 10 (10.0%)	0	1 / 45 (2.2%)
**GI bleed**	1 / 33 (3.0%)	0	0	0	1 / 44 (2.3%)	0
**Haemorrhage**	0	0	1 / 11 (9.1%)	0	1 / 44 (2.3%)	0
**Pancreatitis**	1 / 33 (3.0%)	0	0	0	1 / 44 (2.3%)	0
**Pulmonary embolism**	0	1 / 35 (2.9%)	0	0	0	1 / 45 (2.2%)
**Stroke**	1 / 33 (3.0%)	0	0	0	1 / 44 (2.3%)	0

Table S6: Cause of death by pre-randomisation choice of immunochemotherapy and treatment allocation.

Cause of death	Bendamustine-rituximab		R-CHOP		Overall	
	Bendamustine-rituximab	Ibrutinib plus rituximab	R-CHOP	Ibrutinib plus rituximab	Immunochemotherapy	Ibrutinib plus rituximab
**MCL**	19 / 59 (32.2%)	21 / 59 (35.6%)	17 / 28 (60.7%)	8 / 21 (38.1%)	36 / 87 (41.4%)	29 / 80 (36.3%)
**MCL with associated COVID-19**	2 / 59 (3.4%)	0	0	0	2 / 87 (2.3%)	0
**Infection**	9 / 59 (15.3%)	8 / 59 (13.6%)	2 / 28 (7.1%)	3 / 21 (14.3%)	11 / 87 (12.6%)	11 / 80 (13.8%)
**COVID-19 infection**	9 / 59 (15.3%)	13 / 59 (22.0%)	2 / 28 (7.1%)	4 / 21 (19.0%)	11 / 87 (12.6%)	17 / 80 (21.3%)
**Cardiac**	8 / 59 (13.6%)	9 / 59 (15.3%)	2 / 28 (7.1%)	1 / 21 (4.8%)	10 / 87 (11.5%)	10 / 80 (12.5%)
**Other cancer**	2 / 59 (3.4%)	3 / 59 (5.1%)	2 / 28 (7.1%)	2 / 21 (9.5%)	4 / 87 (4.6%)	5 / 80 (6.3%)
**Old age/frailty**	1 / 59 (1.7%)	0	0	1 / 21 (4.8%)	1 / 87 (1.1%)	1 / 80 (1.3%)
**Old age/frailty with associated COVID-19**	1 / 59 (1.7%)	1 / 59 (1.7%)	0	0	1 / 87 (1.1%)	1 / 80 (1.3%)
**Dementia**	1 / 59 (1.7%)	1 / 59 (1.7%)	0	0	1 / 87 (1.1%)	1 / 80 (1.3%)
**Renal failure**	1 / 59 (1.7%)	1 / 59 (1.7%)	0	0	1 / 87 (1.1%)	1 / 80 (1.3%)
**Respiratory**	0	0	1 / 28 (3.6%)	0	1 / 87 (1.1%)	0
**Respiratory with associated COVID-19**	0	0	0	1 / 21 (4.8%)	0	1 / 80 (1.3%)
**Tumour lysis syndrome**	1 / 59 (1.7%)	1 / 59 (1.7%)	0	0	1 / 87 (1.1%)	1 / 80 (1.3%)
**Unknown**	2 / 59 (3.4%)	0	0	0	2 / 87 (2.3%)	0
**Cardiovascular**	0	0	0	1 / 21 (4.8%)	0	1 / 80 (1.3%)
**GI bleed**	1 / 59 (1.7%)	0	0	0	1 / 87 (1.1%)	0
**Haemorrhage**	0	0	1 / 28 (3.6%)	0	1 / 87 (1.1%)	0
**Pancreatitis**	1 / 59 (1.7%)	0	0	0	1 / 87 (1.1%)	0
**Parkinson’s disease**	0	0	1 / 28 (3.6%)	0	1 / 87 (1.1%)	0
**Pulmonary embolism**	0	1 / 59 (1.7%)	0	0	0	1 / 80 (1.3%)
**Stroke**	1 / 59 (1.7%)	0	0	0	1 / 87 (1.1%)	0

Table S7: Cause of sudden death by pre-randomisation choice of immunochemotherapy and treatment allocation.

Cause of death	Bendamustine-rituximab		R-CHOP		Overall	
	Bendamustine-rituximab	Ibrutinib plus rituximab	R-CHOP	Ibrutinib plus rituximab	Immunochemotherapy	Ibrutinib plus rituximab
**MCL**	1 / 10 (10.0%)	1 / 13 (7.7%)	1 / 3 (33.3%)	0	2 / 13 (15.4%)	1 / 18 (5.6%)
**Infection**	1 / 10 (10.0%)	1 / 13 (7.7%)	1 / 3 (33.3%)	1 / 5 (20.0%)	2 / 13 (15.4%)	2 / 18 (11.1%)
**COVID-19 infection**	1 / 10 (10.0%)	1 / 13 (7.7%)	0	1 / 5 (20.0%)	1 / 13 (7.7%)	2 / 18 (11.1%)
**Cardiac**	5 / 10 (50.0%)	7 / 13 (53.8%)	1 / 3 (33.3%)	1 / 5 (20.0%)	6 / 13 (46.2%)	8 / 18 (44.4%)
**Old age/frailty with associated COVID-19**	0	1 / 13 (7.7%)	0	0	0	1 / 18 (5.6%)
**Renal failure**	0	1 / 13 (7.7%)	0	0	0	1 / 18 (5.6%)
**Respiratory with associated COVID-19**	0	0	0	1 / 5 (20.0%)	0	1 / 18 (5.6%)
**Unknown**	1 / 10 (10.0%)	0	0	0	1 / 13 (7.7%)	0
**Cardiovascular**	0	0	0	1 / 5 (20.0%)	0	1 / 18 (5.6%)
**Pancreatitis**	1 / 10 (10.0%)	0	0	0	1 / 13 (7.7%)	0
**Pulmonary embolism**	0	1 / 13 (7.7%)	0	0	0	1 / 18 (5.6%)

Table S8: Death timing

Timing of all-cause mortality. If a participant dies after discontinuing treatment they are off the study pathway.

Timing of death	Bendamustine-rituximab		R-CHOP		Overall	
	Bendamustine-rituximab	Ibrutinib plus rituximab	R-CHOP	Ibrutinib plus rituximab	Immunochemotherapy	Ibrutinib plus rituximab
**Induction treatment**	9 / 59 (15.3%)	8 / 59 (13.6%)	3 / 28 (10.7%)	4 / 21 (19.0%)	12 / 87 (13.8%)	12 / 80 (15.0%)
**Maintenance**	13 / 59 (22.0%)	23 / 59 (39.0%)	7 / 28 (25.0%)	3 / 21 (14.3%)	20 / 87 (23.0%)	26 / 80 (32.5%)
**Follow-up**	15 / 59 (25.4%)	12 / 59 (20.3%)	5 / 28 (17.9%)	7 / 21 (33.3%)	20 / 87 (23.0%)	19 / 80 (23.8%)
**Off study pathway**	22 / 59 (37.3%)	16 / 59 (27.1%)	13 / 28 (46.4%)	7 / 21 (33.3%)	35 / 87 (40.2%)	23 / 80 (28.8%)

Table S9: Second line MCL treatment by pre-randomisation investigator choice of immunochemotherapy and treatment allocation

Second line therapy	Bendamustine-rituximab		R-CHOP		Overall	
	Bendamustine-rituximab	Ibrutinib plus rituximab	R-CHOP	Ibrutinib plus rituximab	Immunochemotherapy	Ibrutinib plus rituximab
BTK inhibitor	23 / 39 (59.0%)	0	23 / 30 (76.7%)	0	46 / 69 (66.7%)	0
Bendamustine	0	14 / 30 (46.7%)	2 / 30 (6.7%)	1 / 12 (8.3%)	2 / 69 (2.9%)	15 / 42 (35.7%)
CHOP	5 / 39 (12.8%)	3 / 30 (10.0%)	0	5 / 12 (41.7%)	5 / 69 (7.2%)	8 / 42 (19.0%)
R-BAC	0	4 / 30 (13.3%)	1 / 30 (3.3%)	4 / 12 (33.3%)	1 / 69 (1.4%)	8 / 42 (19.0%)
Radiotherapy	5 / 39 (12.8%)	3 / 30 (10.0%)	0	0	5 / 69 (7.2%)	3 / 42 (7.1%)
Pirtobrutinib	2 / 39 (5.1%)	0	2 / 30 (6.7%)	0	4 / 69 (5.8%)	0
High-dose Cytarabine	1 / 39 (2.6%)	1 / 30 (3.3%)	0	1 / 12 (8.3%)	1 / 69 (1.4%)	2 / 42 (4.8%)
Lenalidomide	1 / 39 (2.6%)	1 / 30 (3.3%)	0	0	1 / 69 (1.4%)	1 / 42 (2.4%)
Parsaclisib	0	1 / 30 (3.3%)	1 / 30 (3.3%)	0	1 / 69 (1.4%)	1 / 42 (2.4%)
Venetoclax, Lenalidomide, Rituximab	1 / 39 (2.6%)	1 / 30 (3.3%)	0	0	1 / 69 (1.4%)	1 / 42 (2.4%)
Brexucel CAR T therapy with Ara-C bridging	1 / 39 (2.6%)	0	0	0	1 / 69 (1.4%)	0
R-CVP	0	1 / 30 (3.3%)	0	0	0	1 / 42 (2.4%)
Venetoclax	0	1 / 30 (3.3%)	0	0	0	1 / 42 (2.4%)
MATRIX & Radiotherapy	0	0	1 / 30 (3.3%)	0	1 / 69 (1.4%)	0
Rituximab and chlorambucil	0	0	0	1 / 12 (8.3%)	0	1 / 42 (2.4%)

Table S10: Quality of Life (EORTC QLQ-C30) by pre-randomisation investigator choice of immunochemotherapy and treatment allocation

European Organisation for Research and Treatment of Cancer quality of life questionnaire (EORTC QLQ-C30) scores ranges from 0 to 100, where a higher score represents a higher quality of life. N; median (interquartile range) [min, max] is presented by pre-randomisation investigator choice of immunochemotherapy and treatment allocation by time point.

	Bendamustine-rituximab		R-CHOP		Overall	
	Bendamustine-rituximab N=145	Ibrutinib plus rituximab N=145	R-CHOP N=53	Ibrutinib plus rituximab N=54	Immunochemotherapy N=198	Ibrutinib plus rituximab N=199
**Cycle 1**	133; 84 (73, 91) [29, 100]	137; 87 (68, 94) [27, 100]	47; 86 (72, 95) [24, 100]	51; 83 (75, 94) [38, 100]	180; 85 (73, 93) [24, 100]	188; 86 (69, 94) [27, 100]
**Mid induction treatment**	109; 85 (77, 92) [34, 100]	117; 92 (85, 96) [37, 100]	44; 83 (74, 92) [39, 100]	44; 91 (77, 94) [55, 100]	153; 85 (76, 92) [34, 100]	161; 91 (84, 95) [37, 100]
**End of induction treatment**	102; 89 (78, 93) [53, 100]	107; 91 (81, 97) [25, 100]	42; 84 (71, 95) [13, 100]	46; 90 (83, 96) [30, 100]	144; 88 (77, 94) [13, 100]	153; 91 (82, 97) [25, 100]
**End of maintenance**	60; 90 (83, 96) [44, 100]	65; 91 (86, 97) [33, 100]	22; 90 (84, 96) [34, 100]	33; 90 (84, 95) [58, 100]	82; 90 (83, 96) [34, 100]	98; 91 (86, 97) [33, 100]

Table S11: Adverse events of grade 3 and above during induction treatment and maintenance.

Adverse events are reported by MedDRA preferred term and organ system class, presented if occurring in at least 3% of the safety population within any treatment group up to the end of maintenance. The safety population includes all participants who completed at least one cycle of induction treatment.

Number of participants (percentage of safety population) provides the number of participants experiencing at least one grade 3 or above adverse event. The events per participant year refers to the number of grade 3 or above adverse events (may be more than one per participant) divided by the number of participant years up to the end of maintenance.

[1] All adverse events with organ system class "Cardiac disorders" and/or classified as cardiac-related.

[2] All adverse events classified as bleeding-related.

[3] All grade 3 and above corona virus infections reported refer to COVID-19

**Organ system class**<br>*MedDRA preferred term*	**Bendamustine-rituximab**<br>N=143<br>Years exposed = 245.8	**R-CHOP**<br>N=52<br>Years exposed = 86.8	**Ibrutinib plus rituximab**<br>N=198<br>Years exposed = 360.3
	Number of participants (percentage of safety population), events per participant year		
**Total AEs[1]**	99 (69%), 0.952	37 (71%), 1.059	132 (67%), 0.960
**All cardiac AEs[2]**	9 (6%), 0.053	8 (15%), 0.115	47 (24%), 0.203
**All bleeding AEs[3]**	3 (2%), 0.016	3 (6%), 0.046	11 (6%), 0.039
**Blood and lymphatic system disorders**	39 (27%), 0.273	18 (35%), 0.357	30 (15%), 0.161
Anaemia	4 (3%), 0.020	3 (6%), 0.046	6 (3%), 0.019
Febrile neutropenia	7 (5%), 0.033	5 (10%), 0.069	3 (2%), 0.008
Neutropenia	27 (19%), 0.187	11 (21%), 0.173	18 (9%), 0.092
Thrombocytopenia	7 (5%), 0.028	2 (4%), 0.069	9 (5%), 0.033
**Cardiac disorders**	7 (5%), 0.037	6 (12%), 0.081	42 (21%), 0.180
Atrial fibrillation	1 (1%), 0.008	0	13 (7%), 0.042
Hypertension	2 (1%), 0.008	2 (4%), 0.023	22 (11%), 0.092
Myocardial infarction	1 (1%), 0.004	2 (4%), 0.023	2 (1%), 0.006
**Gastrointestinal disorders**	10 (7%), 0.049	4 (8%), 0.046	11 (6%), 0.047
Diarrhoea	4 (3%), 0.016	0	3 (2%), 0.011
**General disorders and administration site conditions**	10 (7%), 0.053	2 (4%), 0.023	12 (6%), 0.042
Pyrexia	6 (4%), 0.024	1 (2%), 0.012	6 (3%), 0.019
**Infections and infestations**	35 (24%), 0.228	14 (27%), 0.288	55 (28%), 0.225
Corona virus infection	10 (7%), 0.069	1 (2%), 0.012	14 (7%), 0.044
Infection	4 (3%), 0.020	0	2 (1%), 0.006
Lower respiratory tract infection	4 (3%), 0.016	2 (4%), 0.023	5 (3%), 0.019
Lung infection	4 (3%), 0.016	0	1 (1%), 0.003

Neutropenic sepsis	2 (1%), 0.008	8 (15%), 0.138	6 (3%), 0.022
Pneumonia	8 (6%), 0.041	3 (6%), 0.035	11 (6%), 0.039
Sepsis	3 (2%), 0.012	3 (6%), 0.035	8 (4%), 0.022
**Injury, poisoning and procedural complications**	11 (8%), 0.045	4 (8%), 0.081	4 (2%), 0.011
Fall	2 (1%), 0.008	2 (4%), 0.023	0
Infusion related reaction	2 (1%), 0.008	3 (6%), 0.046	1 (1%), 0.003
**Investigations**	14 (10%), 0.073	5 (10%), 0.069	9 (5%), 0.025
Lymphocyte count decreased	5 (3%), 0.037	1 (2%), 0.012	0
**Metabolism and nutrition disorders**	10 (7%), 0.049	1 (2%), 0.012	12 (6%), 0.047
**Musculoskeletal and connective tissue disorders**	1 (1%), 0.004	0	6 (3%), 0.025
**Neoplasms benign, malignant and unspecified (incl cysts and polyps)**	6 (4%), 0.024	1 (2%), 0.012	10 (5%), 0.031
**Nervous system disorders**	4 (3%), 0.016	3 (6%), 0.046	6 (3%), 0.017
**Respiratory, thoracic and mediastinal disorders**	7 (5%), 0.037	2 (4%), 0.023	16 (8%), 0.050
Dyspnoea	4 (3%), 0.020	1 (2%), 0.012	2 (1%), 0.006
Pleural effusion	2 (1%), 0.008	1 (2%), 0.012	6 (3%), 0.019
**Skin and subcutaneous tissue disorders**	9 (6%), 0.037	0	10 (5%), 0.028
Rash	5 (3%), 0.020	0	2 (1%), 0.006
**Surgical and medical procedures**	0	1 (2%), 0.012	6 (3%), 0.019

Table S12: Treatment discontinuation by pre-randomisation investigator choice of immunochemotherapy and treatment allocation

Reasons for active discontinuation of treatment (excluding the treatment stopping events of death and withdrawal)

Reason for discontinuation	Bendamustine-rituximab		R-CHOP		Overall	
	Bendamustine -rituximab	Ibrutinib plus rituximab	R-CHOP	Ibrutinib plus rituximab	Immunochemotherapy	Ibrutinib plus rituximab
**Allergic reaction**	5 / 66 (7.6%)	0	0	0	5 / 94 (5.3%)	0
**Bleeding/haemorrhage**	0	1 / 80 (1.3%)	0	0	0	1 / 104 (1.0%)
**Cardiac**	1 / 66 (1.5%)	13 / 80 (16.3%)	0	2 / 24 (8.3%)	1 / 94 (1.1%)	15 / 104 (14.4%)
**Cardiac/haemorrhage (epistaxis)**	0	1 / 80 (1.3%)	0	0	0	1 / 104 (1.0%)
**Colitis (GI toxicity)**	0	1 / 80 (1.3%)	0	0	0	1 / 104 (1.0%)
**Comorbidities or frailty**	2 / 66 (3.0%)	3 / 80 (3.8%)	0	0	2 / 94 (2.1%)	3 / 104 (2.9%)
**CVA**	0	1 / 80 (1.3%)	0	0	0	1 / 104 (1.0%)
**Cytopenia**	5 / 66 (7.6%)	1 / 80 (1.3%)	0	1 / 24 (4.2%)	5 / 94 (5.3%)	2 / 104 (1.9%)
**Dermatological/pt choice**	0	1 / 80 (1.3%)	0	0	0	1 / 104 (1.0%)
**Electrolyte disturbance**	0	1 / 80 (1.3%)	0	0	0	1 / 104 (1.0%)
**Fatigue**	0	1 / 80 (1.3%)	0	0	0	1 / 104 (1.0%)
**Gastro cause**	2 / 66 (3.0%)	2 / 80 (2.5%)	0	0	2 / 94 (2.1%)	2 / 104 (1.9%)
**Gastro cause (inflammatory bowel disease)**	0	0	0	2 / 24 (8.3%)	0	2 / 104 (1.9%)
**Hypogammaglobulinemia**	1 / 66 (1.5%)	0	0	0	1 / 94 (1.1%)	0
**Infection**	4 / 66 (6.1%)	2 / 80 (2.5%)	1 / 28 (3.6%)	0	5 / 94 (5.3%)	2 / 104 (1.9%)
**Infection (COVID-19)**	3 / 66 (4.5%)	3 / 80 (3.8%)	0	0	3 / 94 (3.2%)	3 / 104 (2.9%)
**Infection (respiratory)**	1 / 66 (1.5%)	1 / 80 (1.3%)	1 / 28 (3.6%)	2 / 24 (8.3%)	2 / 94 (2.1%)	3 / 104 (2.9%)

**Infections/cytopenia**	1 / 66 (1.5%)	0	0	0	1 / 94 (1.1%)	0
**Intolerance (multiple AEs) investigator choice**	1 / 66 (1.5%)	0	0	0	1 / 94 (1.1%)	0
**Neurological**	1 / 66 (1.5%)	1 / 80 (1.3%)	0	0	1 / 94 (1.1%)	1 / 104 (1.0%)
**Treatment for Sarcoidosis**	0	1 / 80 (1.3%)	0	0	0	1 / 104 (1.0%)
**Patient choice**	1 / 66 (1.5%)	3 / 80 (3.8%)	1 / 28 (3.6%)	1 / 24 (4.2%)	2 / 94 (2.1%)	4 / 104 (3.8%)
**Poor response, no documented progression**	4 / 66 (6.1%)	0	4 / 28 (14.3%)	0	8 / 94 (8.5%)	0
**Progressive Disease**	22 / 66 (33.3%)	27 / 80 (33.8%)	20 / 28 (71.4%)	14 / 24 (58.3%)	42 / 94 (44.7%)	41 / 104 (39.4%)
**Respiratory**	3 / 66 (4.5%)	6 / 80 (7.5%)	0	0	3 / 94 (3.2%)	6 / 104 (5.8%)
**Risk of COVID-19 (investigator decision)**	5 / 66 (7.6%)	1 / 80 (1.3%)	0	0	5 / 94 (5.3%)	1 / 104 (1.0%)
**Second malignancy**	3 / 66 (4.5%)	5 / 80 (6.3%)	1 / 28 (3.6%)	0	4 / 94 (4.3%)	5 / 104 (4.8%)
**Subdural haematoma**	0	0	0	1 / 24 (4.2%)	0	1 / 104 (1.0%)
**Thrombosis**	1 / 66 (1.5%)	0	0	0	1 / 94 (1.1%)	0
**Urological**	0	3 / 80 (3.8%)	0	1 / 24 (4.2%)	0	4 / 104 (3.8%)
**Urological/infection**	0	1 / 80 (1.3%)	0	0	0	1 / 104 (1.0%)

Missing data

Progression-free survival and overall survival data were available for all participants. Dates of progression and death were verified by the study team in collaboration with site investigators. Participants without events (including those who withdrew consent to follow-up) were censored at the last known progression-free (or survival) date. Further details of the follow-up schedule can be found in the protocol and statistical analysis plan.

Baseline characteristics, including MIPI, TP53 mutation status, blastoid status and Ki67 had missing values for some participants (Table 1). Following Sterne et al. [1], we performed multiple imputation by chained equations as a sensitivity analysis for the subgroups of MIPI and Ki67, as these variables were considered to have missing data that was plausibly missing at random. Twenty copies of the data set with imputed values were created using the outcome, treatment allocation, chemotherapy choice, MIPI, TP53, blastoid, Ki67, age category, sex, disease stage and ECOG. Results from the data sets were pooled using Rubin's rule [2, 3] and presented below.

Table S13: Subgroup analysis of progression-free survival with multiple imputation of baseline characteristics MIPI and Ki67. The complete case results are from the subgroup analysis in Figure 4.

	**Subgroup analysis of progression-free survival HR (95% CI) Ibrutinib plus rituximab / Immunochemotherapy**	
	**Complete cases**	**Multiple imputation**
**MIPI Low**	0.62 (0.25, 1.53)	0.59 (0.23, 1.56)
**MIPI intermediate**	0.52 (0.30, 0.89)	0.51 (0.29, 0.89)
**MIPI high**	0.77 (0.55, 1.08)	0.77 (0.54, 1.08)
**Ki67 <30%**	0.58 (0.36, 0.94)	0.61 (0.40, 0.94)
**Ki67 ≥ 30%**	0.86 (0.55, 1.34)	0.83 (0.55, 1.25)

[1] Sterne JAC, White IR, Carlin JB et al (2009) Multiple imputation for missing data in epidemiological and clinical research: potential and pitfalls. BMJ 338:b2393. https ://doi.org/10.1136/bmj.b2393

[2] Rubin D (1987) *Multiple imputation for nonresponse in surveys*. New York: Wiley.

[3] Stef van Buuren, Karin Groothuis-Oudshoorn (2011). mice: Multivariate Imputation by Chained Equations in R. Journal of Statistical Software, 45(3), 1-67. DOI 10.18637/jss.v045.i03.

Summary of relevant protocol deviations

	Control	Intervention
**Randomisation**		
On steroids at randomisation	1	0
Included without radiologically measured disease on CT	0	1
Randomised with agreed dose reduction	1	0
Without bone marrow trephine	3	4
**Continued in study with stable disease at mid-treatment (control only)**	3	0
**Stopped treatment without AE or progression**		
Patient choice	2	4
Risk of COVID-19	5	1
Poor response (no documented progression and no stable disease at mid-treatment assessment)	4	0
**Withdrawal**		
Investigator decision	6	4
Participant request	5	6
Recorded progressive disease	1	0
Ineligible due to cardiac function	1	0
